# Impacts on Atlantic Killifish from Neurotoxicants: Genes, Behavior, and Population-Relevant Outcomes

**DOI:** 10.1021/acs.est.4c04207

**Published:** 2024-09-17

**Authors:** Janice L. Albers, Lori N. Ivan, Bryan W. Clark, Diane E. Nacci, Rebekah H. Klingler, Adam Thrash, Juan P. Steibel, Natalia Garcia-Reyero Vinas, Michael J. Carvan, Cheryl A. Murphy

**Affiliations:** † Department of Fisheries and Wildlife, Michigan State University, East Lansing, Michigan 48824, United States; ‡ Office of Research and Development, Center for Environmental Measurement and Modeling, Atlantic Coastal Environmental Sciences Division, U.S. Environmental Protection Agency, Narragansett, Rhode Island 02882, United States; § School of Freshwater Sciences, University of Wisconsin-Milwaukee, Milwaukee, Wisconsin 53204, United States; ∥ Biocomputing and Biotechnology, Institute for Genomics, Mississippi State University, Starkville, Mississippi 39759, United States; ⊥ Environmental Laboratory, US Army Engineer Research and Development Center, U.S. Army Corps of Engineers, Vicksburg, Mississippi 39180, United States

**Keywords:** Fundulus heteroclitus, PCB126, mercury, gene sets, hidden Markov chain models, fish larvae behavior, individual based model

## Abstract

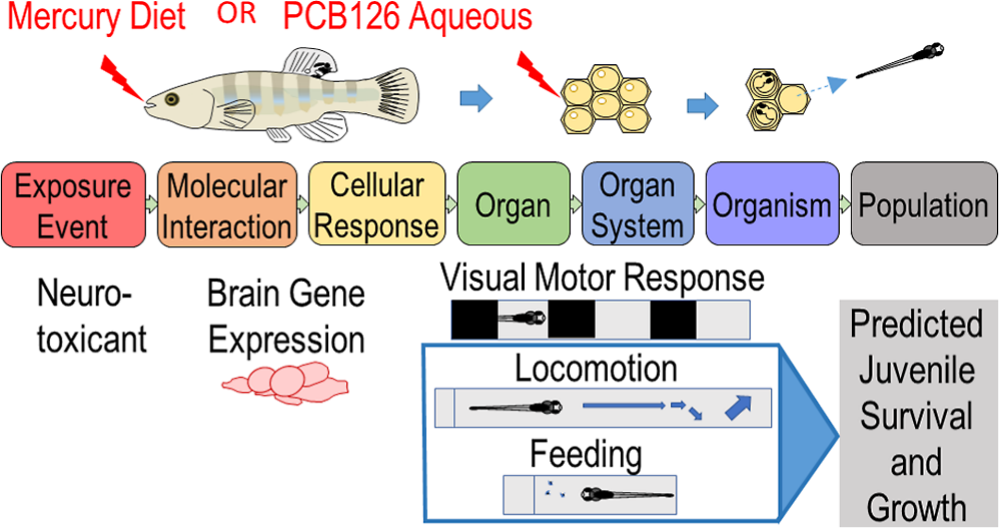

Molecular, cellular, and organismal alterations are important descriptors of toxic effects, but our ability to extrapolate and predict ecological risks is limited by the availability of studies that link measurable end points to adverse population relevant outcomes such as cohort survival and growth. In this study, we used laboratory gene expression and behavior data from two populations of Atlantic killifish *Fundulus heteroclitus* [one reference site (SCOKF) and one PCB-contaminated site (NBHKF)] to inform individual-based models simulating cohort growth and survival from embryonic exposures to environmentally relevant concentrations of neurotoxicants. Methylmercury exposed SCOKF exhibited brain gene expression changes in the si:ch211–186j3.6, si:dkey-21c1.4, scamp1, and klhl6 genes, which coincided with changes in feeding and swimming behaviors, but our models simulated no growth or survival effects of exposures. PCB126-exposed SCOKF had lower physical activity levels coinciding with a general upregulation in nucleic and cellular brain gene sets (BGS) and downregulation in signaling, nucleic, and cellular BGS. The NBHKF, known to be tolerant to PCBs, had altered swimming behaviors that coincided with 98% fewer altered BGS. Our models simulated PCB126 decreased growth in SCOKF and survival in SCOKF and NBHKF. Overall, our study provides a unique demonstration linking molecular and behavioral data to develop quantitative, testable predictions of ecological risk.

## Abstract 2

Links between the environment, gene expression and larval behavior were used to assess impacts from neurotoxicants.

## Introduction

Sublethal levels of neurotoxic chemicals such as polychlorinated biphenyl (specifically 3,3′,4,4′,5-pentachlorbiphenyl congener, PCB126) and methylmercury (MeHg) commonly exist in an industrial landscape as aquatic pollutants.^1^ However, there is a limited ability to predict sublethal impacts or assess risk from these neurotoxic chemicals on individual fish, multiple species, or their populations. One approach to solving this problem is to examine the neurobehavioral impacts through an adverse outcome pathway (AOP) framework^2^ constructed using standard laboratory fish species along with local species of conservation concern. Fundamental to the AOP framework is connecting the chain of events from toxic exposure, molecular initiating event/s, to key events in cellular, organ, and organ systems; to whole organism and/or population level impacts.^3^ Adverse Outcome Pathways are constructed to be modular and chemically agnostic, where comparing the results from two different chemicals can illustrate areas of commonality but also differences (https://aopwiki.org/). For example, PCB126 and MeHg could potentially interrupt different neurological development pathways;^4−7^ consequently, similarities between these two chemicals at the molecular level may be limited. However, similarities may increase as impacts are scaled up from molecular to more integrative organisms and population-level effects.

This study developed an AOP that starts at a neurotoxicant embryonic exposure and measured three key events (i.e., types of end points): (1) brain gene expression, (2) individual behavior, and (3) predicted cohort impacts. Using an AOP framework, these particular key events allow us to elucidate how environmental contaminants influence genes, which in turn influence individual fish behavior.^8^ This research is possible because of recent advances in efficient gene expression/quantification tools, and the AOP framework shows promise in connecting the environment to animal behavior,^9^ especially in toxic environments. Additionally, scientists are gradually organizing studies and conducting research to find new methods in order to use lower levels of biological organization to predict population level impacts (e.g.,^10−12^).

Populations of nonmigratory small *Fundulus heteroclitus* (Atlantic killifish, KF) are a well-known example of a fish species that demonstrates genetic modifications due to a toxic environment. Some populations of KF have persisted in estuaries along the Atlantic coast even after long-term exposure to industrial pollution.^13^ This model fish species is of interest to toxicologists because some populations have been found to have genetically adapted in the wild to dioxin-like contaminants (DLCs)^14^ and other populations continue to persist in mercury-polluted environments.^15−17^ This study examined this unique species and compared two genetically distinct populations, one known to have chemical tolerance and one without ancestral exposure to pollutants. Response differences between these two populations could lead to insight into the molecular machinery underlying this evolved tolerance. Further, examining similarities between gene expression and behavior end points could indicate how brain gene expression drives behavior. Consequently, this study determined differences of brain gene expression, behavior, and cohort metrics after sublethal embryonic exposure to two neurotoxicants, MeHg and PCB126, in adapted and nonadapted KF populations.

## Materials and Methods

### Populations

In this study, two populations of KF were used to assess the effects of a model DLC, PCB126. These KF populations had been found previously to be relatively PCB-sensitive (Scorton Creek, Barnstable, MA; SCOKF) or PCB-tolerant (New Bedford Harbor, MA; NBHKF), respectively.^18^ Disparities in MeHg sensitivity between these KF populations have not been previously documented;^15^ consequently, a subset of the SCOKF population was used to assess effects of MeHg exposure.

### Parental KF Husbandry and MeHg Exposure

Killifish (100–200 fish) were collected from the wild using baited traps and maintained as previously described.^18^ In brief, KF were returned to US Environmental Protection Agency (EPA) Office of Research and Development marine aquarium facilities (Narragansett, RI) and held in ∼250 L tanks supplied with free-flowing, uncontaminated seawater. Relatively uncontaminated SCOKF (parental generation, P) was held in the lab for more than six months before use as breeding stock in this study. However, highly contaminated NBH killifish were held for at least 2 years depuration before producing F1 progeny, which were grown to maturity (1–2 years) and then used as breeding stock in this study. All procedures using live vertebrate animals at the EPA were conducted in accordance with Animal Care and Use Protocols approved by the University of Wisconsin at Milwaukee Institutional Animal Care and Use Committee (IACUC, #18-19#04) and EPA ACUP # Eco23-03002 and Eco230-07-001.

Before the onset of the adult KF dietary exposures (24 April 2017), selected KF were transferred to six ∼250 L tanks (2 NBHKF F1 tanks, 4 SCOKF P tanks), acclimated up to 23 °C (breeding temperature), and then held for 4 weeks. Each tank held 36 (24 female and 12 male) size-matched KF [each individual ∼7 g of mean wet weight (ww) or 1.75 g of mean dry weight (dw)]. KF were fed constructed diets containing ∼30% wild fish (w/w) and components such as Tetramin Tropical Flake, which supported healthy growth and reproduction in KF (unpublished data). The diets included wild sockeye salmon *Oncorhynchus nerka* fillet (naturally low in MeHg) or wild tuna steak (naturally high in MeHg). Because we were unable to obtain any fresh tuna low enough in MeHg to serve as an appropriate control diet, salmon was the best available alternative (^18b^ unpublished data, Kate Buckman, Dartmouth College). Although these diets are very similar nutritionally, there may be aspects of the tuna-based diet other than MeHg that may cause possible differences detected by the study end points and consequently be grouped in the Scorton Creek KF control versus MeHg comparisons. Regardless, a tuna-based diet was used to produce high MeHg KF breeding stock, and a salmon-based diet was used to produce low MeHg or reference (control) KF breeding stock since native whole SCOKF contains a low level of mercury [Hg; 186.10 ± 23.30 ng tHg/g dw KF, standard deviation (SD), *n* = 5, sampled April 27, 2017. The low MeHg KF breeding stock received a daily estimated dose of ∼300 ng tHg/g dw KF/day through their salmon-based diet. Adult KF in this treatment had a body concentration of Hg similar to the wild caught fish referenced above at 162.46 ± 20.21 ng tHg/g dw KF (SD, *n* = 8); their larval progeny contained 9.80 ± 2.49 ng tHg/g dw KF at 3 days post fertilization (dpf, SD, *n* = 9). The high MeHg KF breeding stock received a daily estimated dose of ∼3600 ng tHg/g dw KF/day through their tuna diet. Adult KF in this treatment had a body concentration of 564.09 ± 269.29 ng tHg/g dw KF (SD, *n* = 5); their larval progeny contained 35.09 ± 17.06 ng tHg/g dw KF (SD, *n* = 16) at 2 dpf. Preliminary data (unpublished, Kate Buckman, Dartmouth College) demonstrated that maternal KF achieved tHg concentrations equivalent to their dietary consumption of ∼1200 ng Hg/g dw by day 42 and produced embryos containing 35–100 ng Hg/g dw.

### Treatment Groups of Embryos from KF Breeding Stock

**Figure 1:**
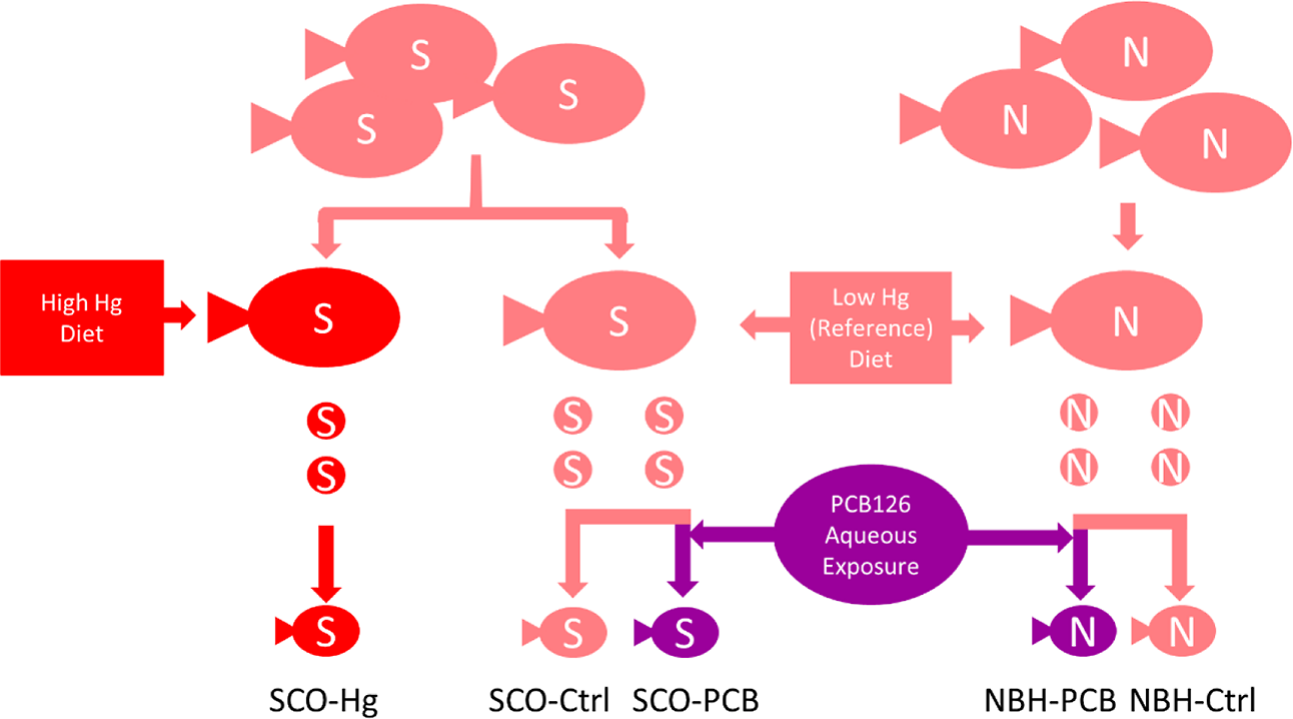
Atlantic killifish larval treatment groups (labeled as in text) showing adult populations from Scorton Creek (S) or New Bedford (N) and fed diets low (control) or high in mercury (Hg), producing embryos (circles), subsets of which were exposed during development to PCB126.

After adult KF dietary exposures (≥103 days) were completed, KF were strip spawned and mixed to produce embryos from each of these three KF breeding stocks: SCOKF low MeHg diet, SCOKF high MeHg diet, and NBHKF low MeHg diet. NBHKF larvae were not tested for higher level MeHg impacts in this experiment because it was outside the scope of the study. Embryos were maintained during early development at the EPA as per the KF embryo larval assay (ELA) protocol, as described below. Subsamples of the embryos from SCOKF low MeHg diet and NBHKF low MeHg diet KF were exposed directly to PCB126 during development, 1 to 7 dpf. Direct exposures to 40 or 400 ng/L nominal concentrations of PCB126 were selected to produce embryo concentrations equal to 0.1× or 1.0×, respectively, to PCB126 levels measured in wild NBHKF (189 ng/g dw).^14^ However, the higher exposure was completely lethal to SCOKF embryos and produced some lethality in NBHKF embryos (Table S1); therefore, these treatment groups were not assessed for behavior. Since Hg tissue concentrations in the low MeHg diet (salmon) were similar to wild caught SCOKF (see concentrations stated in the previous section), the low MeHg diet was labeled as the control. Thus, there were five embryo treatment groups analyzed in the behavior sections of this study (Figure 1). (1) SCOKF embryos from low MeHg diet adult KF without further direct exposures (SCO-Ctrl); (2) SCOKF embryos from high MeHg diet adult KF without further direct exposures (SCO-MeHg); (3) SCOKF embryos from low MeHg diet adult KF with aqueous embryonic exposure to a low level (40 ng/L) of PCB126 (SCO-PCB); (4) NBHKF embryos from low MeHg diet adult KF without further direct exposures (NBH-Ctrl); and (5) NBHKF embryos from low MeHg diet adult KF with aqueous embryonic exposure to a low level (40 ng/L) of PCB126 (NBH-PCB). Of all the possible pairwise comparisons between the five treatments, this study was focused on only three types of comparisons. (1) The comparison that determined only high MeHg impacts, SCO-Ctrl vs SCO-MeHg. (2) The four comparisons between the PCB treatments and KF populations [(a)SCO-Ctrl vs SCO-PCB, (b) SCO-Ctrl vs NBH-Ctrl, (c) SCO-PCB vs NBH-PCB, and (d) NBH-Ctrl vs NBH-PCB]. (3) All five of these comparisons combined to determine if there were any responses that were similar between the two chemicals.

### Embryo-Larval Assessments

Routine rearing and monitoring of the early development of KF embryos, ELA methods, were conducted as previously described.^19^ Briefly, one dpf embryos were transferred into individual vials containing 10 mL of seawater, amended with acetone (0.01% acetone, Sigma Chemical, St. Louis, MO, USA) or chemical-acetone stocks of PCB126 (Accustandard, New Haven, CT). At seven dpf, embryos were transferred to a 12-well disposable plate (Thermo Fisher Scientific, Rockville, MD, USA) containing uncontaminated seawater-dampened 20 mm Restek Cellulose filters made for ASE 200 extraction cells (Restek, Bellefonte, PA, USA). A subset of embryos from each treatment group were sent to UWM for Hg or behavioral analyses. The remaining embryos from each treatment group remained at EPA (>20) and incubated at 23 °C. At 10 dpf, embryos were phenotyped microscopically for abnormalities in developmental stage, and features were noted.^20,21^ At 14 dpf, seawater was added to each well, and the plates were rocked gently to initiate hatching. Individual larvae were maintained in single wells containing 3 mL of seawater for all assessments, incubated at 23 °C, fed 24 h hatched Artemia ad lib daily, and renewed with seawater on alternate days. Individuals were assessed daily for survival until 7 days post hatching (dph) when the ELA was terminated (Table S2).

To assess the degree of neurological impact, three different larval behavior assays were conducted on larvae that showed no external signs of physical abnormalities: a visual motor response assay (VMR), a free-swimming locomotion assay, and a feeding assay (Figure S1). From these assays, 83 different larval behaviors were measured, resulting in 48, 30, and 5 end points per assay, respectively. See the Supporting Information Section for behavior assay details. Location data for each embryo in each assay can be found at datadryad.org (10.5061/dryad.12jm63z6w).

#### Behavior/Gene Expression Comparisons

Larval behaviors and brain gene expression were determined using methods outlined in the Supporting Information (page 1–7). In brief, each end point response, either gene or behavior, was summarized over all treatments by first determining whether there was a significant difference found while testing the treatment comparisons using Bayesian modeling. By random chance, the number of behavior tests that could be significant is ∼4 (0.05 alpha level × 83 tests = 4.15). When a significant difference was found, a positive (Pos) or negative (Neg) trend was used to indicate the relative response amount of the second treatment to the first treatment. For example, in the comparison between SCO-Ctrl vs SCO-PCB treatments, if the SCO-PCB treatment had a higher level than the SCO-Ctrl treatment, the summary is positive. If the SCO-PCB treatment had a lower level than the SCO-Ctrl treatment, the summary is negative. The end point value used to determine the trend direction was the back-transformed treatment means. A similar process was used to determine brain gene expression patterns. Trends in gene expression were determined by comparing treatment and control groups using negative binomial models and a false discovery rate of 0.05.

The resulting trend patterns from both sets of tests were used to group behavior and gene expression end points that responded the same. This approach is more robust than other data mining methods (e.g., PCA) because it (1) takes into account the treatment design of the experiment and the comparisons and (2) limits comparisons to only those end points that were determined to be statistically different from one another, which limits the excessive comparison of all end points.

#### Individual-Based Model

A generalized individual-based model (IBM) was developed that simulated the sublethal effects of MeHg and PCB126 on the two different populations of larval KF and predicted the impacts of simulated growth and survival from the larval to juvenile stages of development. The model is described in a previous work,^22^ with brief details provided here. The IBM was adapted from a generalized larval fish model^23^ using bioenergetic equations from the California killifish *Fundulus parvipinnis*.^24^ It included five larval fish behaviors that were observed in the laboratory tests to simulate larval to juvenile food consumption. Multipliers were placed on larval swimming speed derived from the locomotion assay, larval capture success of zooplankton, larval handling time of zooplankton, and larval reactive distance to zooplankton derived from the feeding assay. The IBM-tracked 2500 individual larvae from hatch to juvenile stage or until 100 days,^25^ whichever occurred first (Figure S2). For each scenario (population × toxicant effect), the model was run 10 times to account for individual stochasticity. All results are reported as means of each simulation within a scenario. Due to the potential for KF spring spawning to be extended into the summer and the impact of higher temperatures on growth and survival, the model tracked larvae during two time periods (spring or summer; see Supporting Information Section for more details). Since model uncertainty did not affect the relative comparisons between controls and treatments for KF in the previous IBM study,^22^ the model used in this study did not contain parameter uncertainty, only individual fish stochasticity. See Supporting Information for more details, and Table S11 for all model parameter values.

## Results

### Behavior End Points

Many larval behaviors were affected by MeHg in their parent’s diet and/or aqueous exposure to PCB126 during development. Of the 83 behavior end points tested, 49 had at least one treatment difference from chemical exposure (Tables S14, S15, S21–S23). More significant behavior patterns were found with behavior end points that examined swimming characteristics (30) than were found with stamina/activity type behaviors (24), even though both had similar levels of testing over all assays (33 total swimming characteristics were examined, 31 stamina/activity type behaviors, 2 startles, 5 feeding behavior types). Although both the VMR and locomotion assay were examined for the same suite of 10 swimming end points (Table S3), the same set of swimming end points did not exhibit the same trends across treatments (Tables S21 and S22). The exceptions being (1) the overall swimming bout turning angle from the locomotion assay and the average swimming bout turning angle in light periods 2 and 4 of the VMR assay (Table S21, ref (2)); and (2) swimming bouts (per sec) and the number of swimming bouts per second in periods 2–5 in the VMR assay (Table S21, ref (3)). End points such as swimming bout duration, total time swimming, and total distance traveled did not consistently report treatment differences during lighted or dark periods in the VMR assay and the locomotion assay.

Increased parental MeHg exposure altered SCOKF larvae behavior end points (Table S22). For example, SCOKF larval capture probability, capture attempt ratio, and reaction distance increased when higher MeHg levels were fed to their parents. Mercury also increased the probability of a SCOKF larva staying in the fast or medium swimming state. In addition, MeHg exposure decreased SCOKF larva total distance traveled, step length, and variation in the final VMR period; swimming bout duration and total time swimming in the VMR period 3; turning angle variation in the medium behavior state; and the transition probability from the medium to fast state (Table S22).

Occasionally both MeHg and PCB126 made certain behavior end points respond similarly in the SCOKF larvae (Table S23). For example, both MeHg and PCB126 made SCOKF larvae increase the number of capture attempts, with PCB126 increasing it more than MeHg (Table S23, ref (2)). Additionally, both chemicals decreased the duration of swimming bouts and total time swimming in VMR period 3 (Table S23, refs (1 and 3)).

Most behavior alterations found in this study were from exposure to PCB126 during larval development (Table S21). For example, larval handling time of prey increased in every PCB126 treatment (Table S21, ref (1)). Other behavior end points increased with PCB126 exposure but differed in severity between populations, such as SCOKF larvae proportionally missing more prey, but NBHKF larvae missed even more (Table S21, ref (2)). Swimming bouts during light periods also changed with increases in the turning angle (Table S21, ref (2)) and decreases in bout frequency (Table S21, ref (3)) both in the locomotion and VMR assays; again more severely in the NBHKF larvae.

Some behavior end points were only altered by PCB126 in either the SCOKF or NBHKF. For the SCOKF larvae, PCB126 decreased SCOKF larvae total time swimming (Table S21, ref (7)) and total distance traveled in the locomotion assay and swimming bout duration in the VMR period 1 (Table S21, ref (5)); total distance traveled, overall step length, and variation in VMR period 3 (Table S21, ref (3)). PCB126 increased SCOKF larvae overall mean turning angle in the VMR period 2 and turning angle variation in period 3 of the VMR, with the latter being higher in the NBHKF larvae but no different than the NBHKF controls (Table S21, refs (2 and 4)). For only the NBHKF larvae, PCB126 decreased the probability of staying in the slow or medium state in addition to the medium to slow state transition probability (Table S21, ref (10)); increased the medium state turning angle; and increased slow to medium, fast to slow, and slow to fast state transition probabilities (Table S21, refs (11, 13 and 15)). Lastly, after PCB126 exposure, NBHKF larvae had a smaller mean and variation in the medium state step length in the locomotion assay, but the NBHKF mean medium step length was still higher than the SCOKF (Table S21, refs (12 and 14)).

Thirteen feeding, swimming, and startle behavior end points were different between the control SCOKF and NBHKF (Table S21, refs (6–11 and 15)). As compared to NBHKF, SCOKF larvae had higher swimming bout duration (Table S21, ref (9)) and total time swimming (Table S22, ref (7)). The SCOKF larvae also had higher transition probabilities from the medium to the fast state (Table S21, ref (9)), slow to medium or fast states (Table S21, refs (11 and 15)). Lastly, SCOKF larvae had higher medium-state turning angles as compared to NBHKF larvae (Table S21, ref (11)). As compared to SCO, NBHKF larvae were higher in larval capture probability, reaction distance (Table S21, ref (8)), and capture attempts (Table S21, ref (6)). The NBHKF larvae also had higher startle magnitude in period 3 of the VMR (Table S21, ref (8)), as well as higher transition probability from slow to slow, medium to slow, and medium to medium state swimming (Table S21, ref (10)).

### Genetic End Points

On average, there were 64,986,960.22 fragments per sample, with a SD of 70,133,05.57. The average mapping rate of the reference transcriptome was 80.49%. Of the 26,771 transcripts quantified, 16,017 transcripts were retained after filtering. The comparison of two groups of fish with the most differentially expressed genes was between the SCO-Ctrl and NBH-Ctrl with 3220 (Table 1). However, SCOKF and NBHKF larvae have only 210 differences in gene expression when both are exposed to a low dose of PCB126 (SCO-PCB 40 ng/L vs NBH-PCB 40 ng/L). SCOKF larvae had 383 altered genes as compared to the controls after exposure to the low PCB126 dose, which is 29 times more than the 8 altered genes found in the NBHKF larvae after low dose PCB126 exposure compared to the control. Even though the NBHKF larvae had few gene alterations after exposure to the low dose PCB126, the high dose of PCB126 altered the gene expression an order of magnitude higher with 830 genes differentially expressed, suggesting that 5% of NBHKF larvae genes are altered by high levels of PCB126. All differentially expressed genes and pathways found in this study are reported in Tables S16 and S17. In addition, all patterns that were found to be similar between differentially expressed genes and behaviors can be found in Tables S18 and S19.

**Table 1:** Total Number of Significantly Differentially Expressed Genes (Alpha = 0.05) Found in the Brains of Atlantic Killifish *F. heteroclitus* in This Study (IBM = Individual Based Model, S = Simulated Larval to Juvenile Survival, G = Simulated Larval to Juvenile Growth, NA = Not Applicable, ND = No Differences Detected, MeHg = Methylmercury, SCO = Scorton Creek, Ctrl = Control Treatment, and PCB = PCB126 Treatment)

treatment comparison	number of differentially expressed genes	number of behavior end point differences	simulated differences from IBM
SCO-Ctrl vs SCO-MeHg	22	12	ND
SCO-Ctrl vs SCO-PCB 40 ng/L	383	24	S and G
SCO-Ctrl vs NBH-Ctrl	3220	13	ND
SCO-PCB 40 ng/L vs NBH-Ctrl	602	NA	NA
SCO-PCB 40 ng/L vs NBH-PCB 40 ng/L	210	23	ND
SCO-PCB 40 ng/L vs NBH-PCB 400 ng/L	1348	NA	NA
NBH-Ctrl vs NBH-PCB 40 ng/L	8	11	S and G
NBH-Ctrl vs NBH-PCB 400 ng/L	830	NA	NA
NBH-PCB 40 ng/L vs NBH-PCB 400 ng/L	896	NA	NA

### Behavior/Gene Expression Comparison

The SCOKF exposed to either MeHg or PCB126 exhibited a change in the number of times the KF larvae attempted to capture prey and the duration of swimming bouts during period 3 of the VMR (Table 2). The same reaction to chemical exposure observed in these two behaviors was also observed in four genes, including the scamp1 gene, which is predicted to be involved in protein transport and degradation of the trans-Golgi network membrane.

**Table 2:**
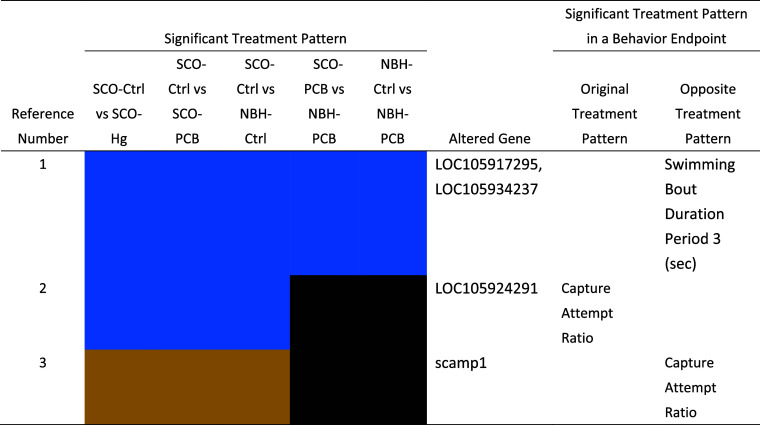
Significant Treatment Patterns from MeHg (Hg) and PCB126 (PCB) Exposure Shared by Genes and Behavior End Points in Scorton Creek (SCO) and New Bedford Harbor (NBH) Atlantic Killifish *F. heteroclitus* Found in This Study. Both the Original and Opposite Behavior End Point Trends are Listed (Ctrl = Control Treatment, Tan = Significant Negative Trend Compared to Control, Blue = Significant Positive Trend Compared to Control, and Black = No Significant Trend Compared to Control)

By itself, the higher dose of MeHg in the SCO parents created offspring that exhibited increased frequency of multiple feeding behaviors such as capture attempt ratio, capture probability, and reaction distance (Figure S5 and Table S18). These increases coincided with the upregulation of 16 genes, including si:ch211-186j3.6, which is thought to be involved with calcium ion binding activity and homophilic cell adhesion. The downregulation of six genes coincided with decreases in five different sustained swimming behaviors in the last two periods of the VMR as well as decreases in medium to fast swimming transition probability detected in the hidden Markov chain model (HMM) analyses. These six genes include si:dkey-21c1.4 (integral component of the membrane), scamp1 and klhl6 (B cell receptor signaling pathway and germinal center formation).

PCB126 exposure resulted in the majority of the alterations found in both the gene expression and behavior. The most changes observed after PCB126 exposure occurred in SCOKF larvae, resulting in the most similarities between gene expression and behavior treatment patterns (Figure 3 and Table S19). Altered brain gene expression mainly occurred with nucleic functions, followed by cellular, signaling, neural, and metabolic functions. These changes coincided with altered stamina swimming type behaviors such as total distance traveled and total time swimming as well as capture attempt ratio. PCB126 also affected NBHKF larvae, but with fewer alterations to gene expression and behaviors. PCB126 down regulated the cmc2 and rab4a genes in NBHKF larvae, resulting in the perturbation of the metabolic KEGG pathway involved in oxidative phosphorylation (KEGG 190; Figure S6 and Table S24). This pathway is important in providing energy and regulating metabolism in the brain and has been connected with multiple neurodegenerative diseases.^26,27^ In addition to these genomic changes, six HMM behaviors were also altered in NBHKF larvae, mainly pertaining to transition probabilities between swimming states.

The two populations of KF had numerous differences in gene expression (3,088), gene sets and pathways (275), and behaviors (11; Tables S19, S20, and S21). The genes that were most different between the two control populations were genes that were involved with nucleic, cellular, and signaling (Table S19), while the gene sets and pathways were involved in nucleic, metabolic, and cellular processes (Table S20). These transcriptomic differences coincided with NBHKF larvae having lower swimming bout duration lengths, higher capture probability, and longer reaction distance as compared to the SCOKF larvae.

After PCB126 exposure, the two populations of KF did have a unique group of gene sets that both were (1) initially expressed differently between the two populations and (2) were altered in SCO larvae but not NBKF larvae (Table S19). A total of 228 different gene sets were associated with this comparison, mainly cellular (29), nucleic (29), signaling (20), neural (18), and metabolic (17) gene sets. These gene expression changes coincided with similar trends in the feeding lung ratio and total time swimming behaviors.

### Individual Based Model

**Figure 2:**
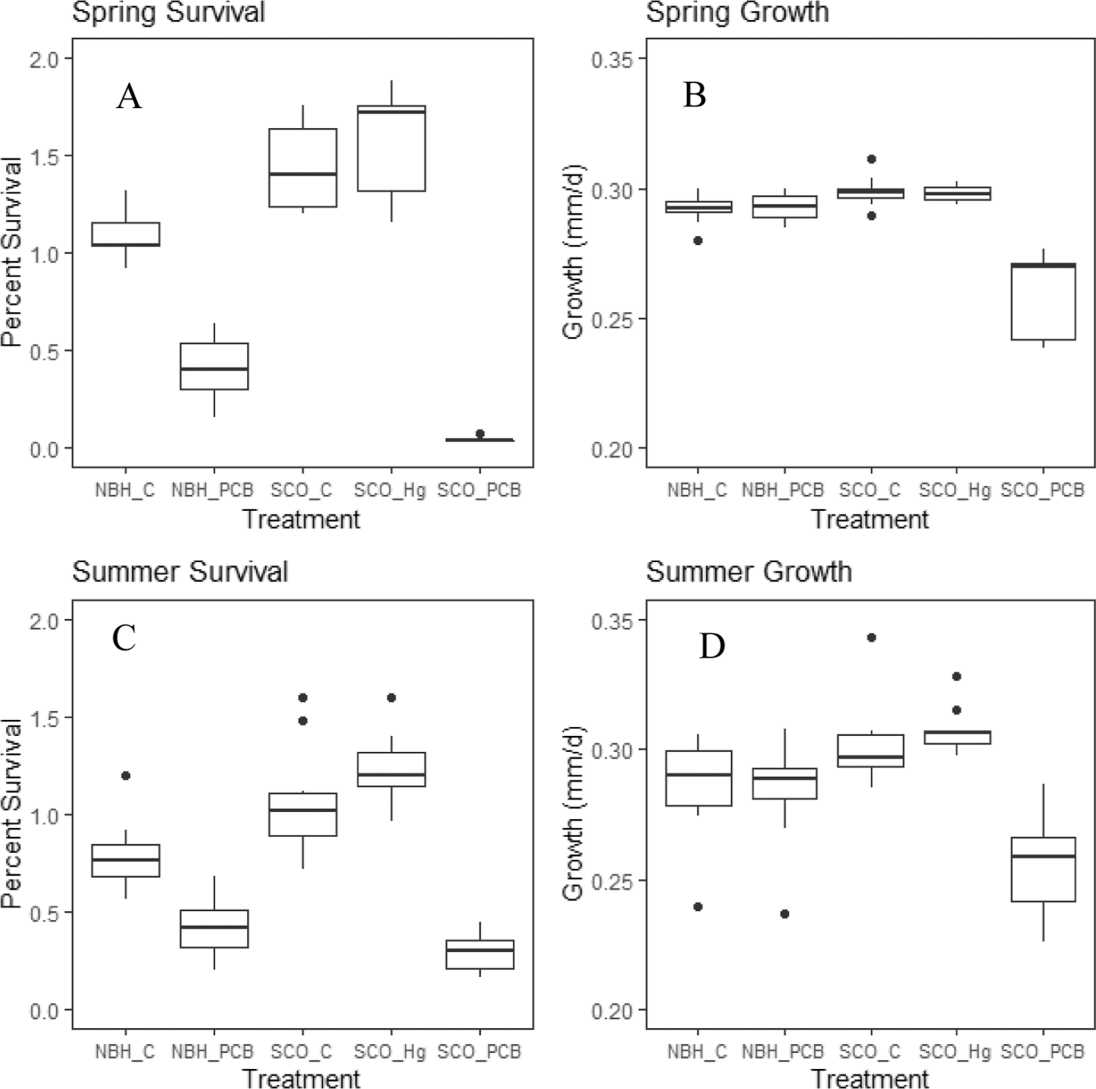
Simulated mean percent survival and growth (mm/d) of Atlantic killifish juvenile survivors for 10 replicates of each treatment for spring and summer scenarios. Presented are box plots showing the mean (bold line), interquartile range (box), 95th percentiles (vertical lines), and 100th percentiles (dots). SCO = Scorton Creek, NBH = New Bedford Harbor, Hg = methylmercury treatment, PCB = PCB126 treatment, and C = control treatment (treatment means reported in Table S25).

The SCOKF and NBHKF simulated cohort growth and survival were different among toxicant treatments. Control cohorts for both SCOKF and NBHKF experienced similar survival rates (1–2%), with SCOKF mean survival 28% higher than that of NBHKF (Figure 2 and Table S21). Likewise, simulated growth rates of the SCOKF control were 2.3% higher than those of the NBHKF control larvae (∼0.3 mm/d; Figure 2A). The effects of MeHg on the SCOKF cohorts were minimal, with MeHg treatment resulting in a 9% higher survival than that of the control (Figure 2). In contrast to MeHg, exposure to PCB126 produced substantial sublethal effects in both SCOKF and NBHKF. SCOKF cohorts exposed to PCB126 as embryos experienced almost no survival in any replicates after 100 days (Figure 2). NBHKF cohorts exposed to PCB126 as embryos had a low survival rate at 0.4% (Figure 2), which was 38% lower than the control. Growth rates between the control and PCB treatments in NBHKF were the same at 0.29 mm/day (Figure 2). Patterns between spring and summer runs were similar in both growth and survival for both populations and treatments. One notable exception was the summer SCOKF cohort that was exposed to PCB126, as embryos ended up with 0.29% higher survival after 100 days as compared to spring, but the growth rate remained 85% less than that of the control (Figure 2).

## Discussion

This study found numerous altered larval gene expressions and behaviors after exposure to MeHg or PCB126 KF as embryos. In addition, multiple altered gene expressions and behaviors changed with the same pattern across the treatments, suggesting an association between the altered gene expression and the performed behavior. Lastly, these altered behaviors resulted in a reduction of the simulated cohort survival of PCB126-exposed KF larvae and reduced cohort growth in SCOKF larvae. The multiple key event alterations found in this study suggest multiple AOPs after the sublethal embryonic exposure of PCB126 or MeHg. In addition, the two different KF populations responded differently to the same PCB126 exposure, suggesting flexibility in KF population response that depended on ancestor exposure history.

Both MeHg and PCB126 exposure produced downregulation in the scamp1 gene and decreases in capture attempts (Table 2). PCB126 and MeHg are contaminants that commonly co-occur in polluted aquatic environments. Multiple AOPs have been identified for each of these neurotoxicants, but it is unclear whether they share any AOPs.^28−31^ Research into each of these neurotoxicants as individuals and in combination has been a long-standing human risk research question since there is the potential for human embryo exposure to both neurotoxicants after contaminated parental fish consumption. Whether MeHg, PCB126, or MeHg + PCB126 antagonize or potentiate impacts during embryo development is still an active research question, generating mixed answers in studies that used rats as test subjects. Results so far indicate that depending on the end point examined, age, or sex of the rat, the combination of MeHg and PCB126 exposure can be additive, synergistic, or dampening (^32−35^ and references therein). However, similarities between MeHg and PCB126 exposure on fish development have only just begun to be assessed, but using fish instead of rats may lead to the same ambiguous answer. Our previous study that examined similar behavior end points in yellow perch (*Perca flavescens*) found no similarities between embryos exposed to either MeHg or PCB126,^36^ which is contrary to the results from this study using KF. However, the gene set responses found between the MeHg and PCB126-exposed KF larvae in this study could be important end points to study when investigating whether these two chemicals work in an additive, synergistic, or dampening way. Comparisons between this study and a future study examining gene sets in larvae that are exposed to a mixture of MeHg and PCB126 may lead to direct determination of the type of chemical mixture interactions.

Mercury-exposed SCO parents produced offspring with altered gene expression and behaviors (Figure S5 and Table S18). These changes involved four known genes involved in signaling, immunity, protein transport, and metabolism that coincided with feeding behaviors, swimming characteristics, and stamina. The klhl6, scamp1, si:ch211-186j3.6, and si:dkey-21c1.4 genes have not been previously reported as mercury-sensitive genes. Although behavior effects from these altered genes are likely since swimming is directly linked to fish metabolic and cell signaling processes. This study is the first to report that these genes had a connection to fish behavior end points. These behavior end points included HMM behaviors (medium swimming state turning angle variation, staying in the fast-swimming state, and transitioning from the medium to fast swimming state), fish larva stamina in the last period of the VMR assay (total distance traveled, step length, and variation), feeding reaction distance, and the probability of capturing prey. These MeHg effects on fish swimming behaviors were expected because MeHg exposure predominately affects the hippocampus region of the brain,^37^ the same region that regulates swimming behavior in fish.^38−40^

The alterations in SCOKF larval gene expression and behaviors after MeHg exposure did not ultimately result in decreases in simulated cohort survival or growth. The IBM in this study predicted MeHg had either no effect or slight increases in cohort survival and growth. The averaged survival of simulated SCOKF in both spring and summer scenarios increased 0.16 or 13% from the control treatment caused by an increase in both capture rates and increases in the distance at which larvae detected prey; increases in these feeding metrics offset the loss in feeding from slower movement rates. This resulted in *a* < 1% change in simulated cohort growth. Previous research has shown that MeHg exposure can increase or decrease fish larvae feeding metrics (see review in ref (41)). This may occur because feeding behavior is a combination of many different physical attributes, such as swimming, perception, and sight. Consequently, the IBM was a good tool to summarize changes to multiple behavior end points into an overall group level change showing an increase in simulated survival and growth.

**Figure 3:**
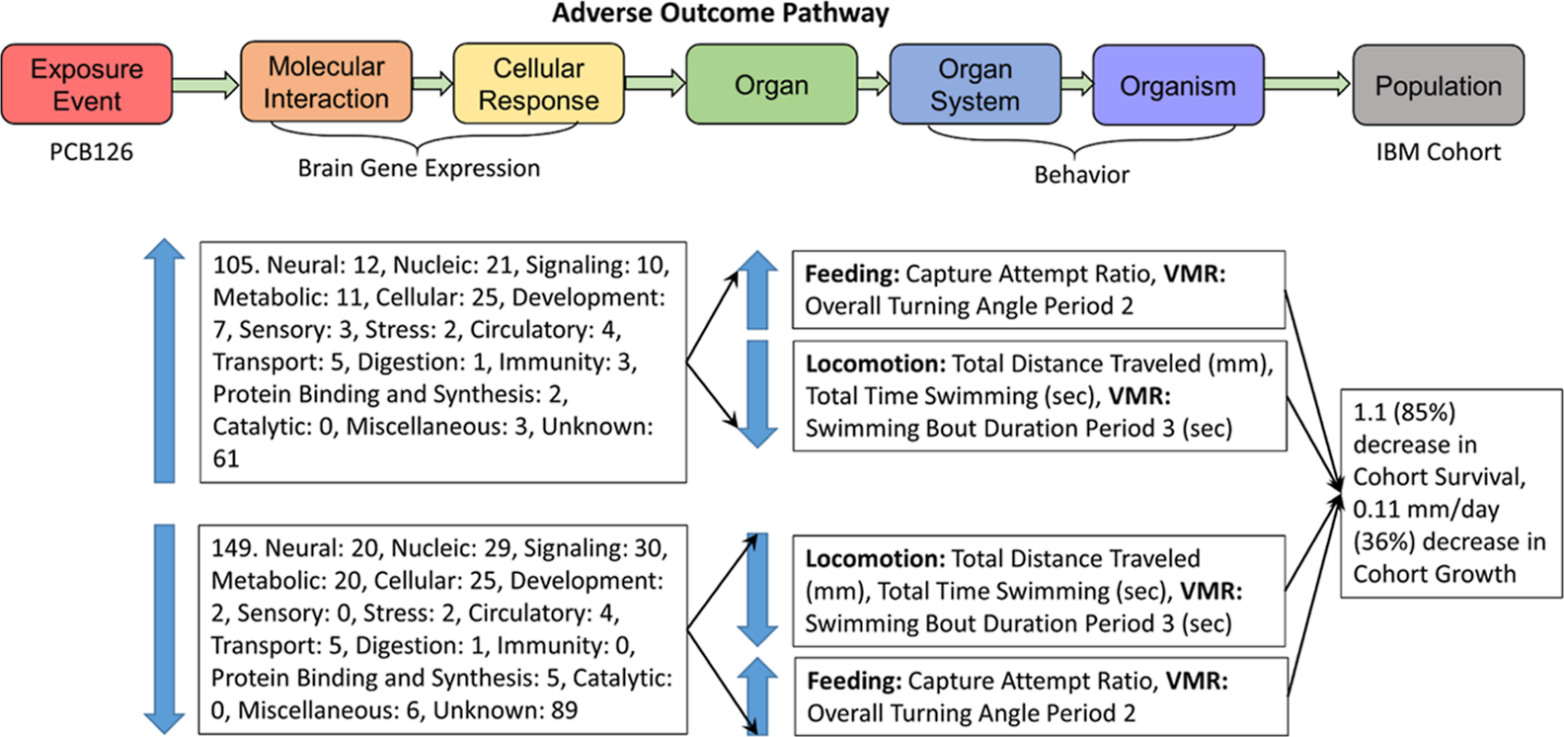
Significant PCB126 response patterns shared by gene expression and behavior end points in the Scorton Creek (SCO) Atlantic killifish found in this study. Both the original and opposite behavior end point trends are listed (VMR = Visual Motor Response assay)

Scorton Creek KF embryos exposed to PCB126 also had altered gene expression and behaviors, linking PCB126 embryonic exposure to both molecular and organism-level effects as well as associating specific behaviors with certain gene expressions (Figure 3 and Table S19). Scorton Creek KF larvae after exposure had lower physical activity levels that were associated with many altered genes and showed a general upregulation in numerous genes involved in nucleic and cellular brain functions and downregulation in signaling and nucleic and cellular functions. Decreases in the total time swimming and total distance traveled were associated with an upregulation of nerve maintenance, development, and neurotransmitters (e.g., genes *lrrc4*.*1*, *atcaya*, *ext2*, and *gad2*), as well as brain ubiquitin processes (e.g., genes *hectd1*, *lnx1*, *neurl1aa*, *rnf41*, *spata2*, *tulp4a*, *uba1*, and *ubap1*) and cellular functions. Additionally, the decrease in activity coincided with a downregulation of brain DNA functions such as binding, splicing, and transcription (e.g., *elk4*, *fam98a*, *kdm2ab*, and *seta*); as well as brain metabolism (e.g., *arfgap1*, *atp8a2*, *elovl6*, and *pitpnab*). Previous research has also found links between PCB126 exposure and decreases in tissue energy supplies and impaired adult fish swimming ability.^42,43^ Aluru et al. (2017) found adult zebrafish exposed to PCB126 as embryos to also have enrichment of calcium signaling and MAPK signaling pathways and downregulation of various metabolic pathways. Other studies found PCB126 embryonic exposure did not alter larval behavior but impaired adult short- and long-term habituation to novel environments,^44^ suggesting that reprogramming gene expression patterns during development could extend impacts into adulthood.

In this study, the SCOKF larvae had the most altered gene expression and behaviors compared to any other group, and this resulted in the highest simulated change in the cohort survival and growth. The behavior changes to SCOKF after exposure to PCB126 resulted in simulations with an 85% decrease in cohort survival, down to 1.1%, and a 36% decrease in growth (0.11 mm/day). These results were from PCB126 having a large impact on SCOKF swimming and travel time as well as handling time. These behavior changes resulted in very low simulated cohort survival in these scenarios, suggesting substantial decreases in the longevity in KF populations without any evolved tolerance. This would ultimately suggest that all exposure levels of PCB126 in this study are lethal to the survival of young-of-year fish. Previous work in zebrafish suggests larval mortality from embryonic exposure to PCB126 because of developmental effects on swim bladder inflation and cartilaginous tissues.^45^ While Glazer et al. (2016) found no effect on zebrafish swimming behaviors, they did find impairment in short- and long-term habituation to a novel environment in adult zebrafish. Multiple molecular alterations have been implicated, including reprograming of brain gene expression patterns resulting in changes in adult brain metabolism and behavior,^46^ as well as the PCB126 altering liver gluconeogenic enzymes in rats leading to wasting disorders.^47^ These larval and cohort effects after embryonic exposure are an important aspect in understanding population trends and risks to population persistence, while individuals of the population are being embryonically exposed to sublethal levels of PCB126.

Although NBHKF larvae were collected from a known PCB-tolerant wild population, the F1 offspring in our study were still affected by PCB126 as determined using behavior and gene expression end points, albeit to a lesser degree than the nontolerant Scorton Creek larvae. New Bedford Harbor KF larvae had subtle swimming characteristics that were altered after PCB126 exposure (Table S24), which coincided with 98% fewer brain gene expression changes as compared to the PCB126 exposed SCOKF larvae (Figure 3 and Table S20). However, with these fewer changes in behaviors and brain gene expression, this study still predicted that NBHKF larva had decreased simulated survival (54% relative to control), although not as extreme as the SCOKF (Figure 2). The decrease in NBHKF simulated survival was from decreased swimming time, resulting in lower encounters with prey relative to the control cohort. Results from this study suggest NBHKF populations are susceptible to low levels of embryonic exposure to PCB126 even with evolved pollution tolerance, which was not seen in lethality and ethoxyresorufin-*O*-deethylase (EROD) activity end points examined in previous studies (see review Nacci et al. 2010). These results suggest NBHKF may have evolved tolerances that allow the population to persist, but this evolved tolerance does not prevent all sublethal impacts from occurring, such as those found in this study. Multiple reasons may exist as to why this study found impacts to NBHKF after PCB126 exposure and not in previous studies. (1) Use of behavior and genetic end points that are in general more sensitive to chemical exposure in fish than lethality or gross morphology.^48−50^ (2) Use of sensitive HMM behavior end points to detect larvae behavior alterations, as compared to traditional behavior end points, where HMM behavior end points have been shown to increase the sensitivity of toxicological behavior analyses.^36^ (3) Examination of all differentially expressed genes in larval brain tissue and not just those genes previously described as being affected by DLCs.

Each of the two KF populations examined in this study responded to PCB126 exposure in unique ways. Killifish offspring from a population with no previously documented exposure to DLCs (SCO) had substantial alterations to their brain gene expression, behavior, and simulated survival and growth after PCB126 exposure. While offspring from a KF population with a known tolerance to DLCs (NBH) were still affected but had different and fewer alterations to their behavior and brain gene expression and not as severe reduction in predicted survival, relative to SCOKF. In comparison to SCOKF larvae, NBHKF larvae appear to have an evolved oxidative phosphorylation pathway (KEGG 190), being already at a lower state before PCB126 exposure relative to SCOKF and only altered in NBHKF after exposure, possibly from their ancestral history with DLCs. These results are not unexpected since previous research suggests KF may be an emerging example of parallel contemporary evolution driven by human-mediated pollution,^51^ especially with DLCs.^18^ The KF ability to adapt seems to be driven by the extremely high genetic variation, especially in genes associated with immune function and olfaction.^13^ Indeed, the highest changes of differentially expressed genes were found when comparing the control groups between NBHKF and SCOKF at 3220 (Table S9). The lowest number of changes was observed between NBHKF control larvae and PCB126 (40 ng/L) treatments with only 8 DEGs. Previous research indicates that KF genes associated with neurological development and cytoskeletal have changed the least, indicating they are required for population persistence.^13^ Results indicate embryonic exposure to PCB126 impacts these same gene types in both the nonadapted and adapted KF, but to a lesser extent in adapted KF.

Even though NBHKF are known to have a tolerance to chemical pollutants, the mechanism of tolerance is yet to be fully understood. Our results suggest that the pollution tolerance may be associated with a metabolic pathway (Table S20), as well as other possible evolved differences due to population isolation. However, previous research into KF tolerance has mainly focused on cytochrome P450 (*Cyp*) and aryl hydrocarbon receptor (*AhR*) gene expression in gill and liver tissues.^18,20,21,51−55^ NBHKF have evolved tolerance by increasing resistance to reactive oxygen species and cardiac teratogenesis^20,54^ mainly through bypassing components in the complex stress response network, which involves *AhR* and *Cyp* gene expression.^51^ The present study did not examine liver or gill tissue, but brain tissue, where *AhR* regulates the timing of restorative neurogenesis and is crucial for the survival of newborn neurons.^56^ Fish brain tissue contains *AhR1* and *AhR2*,^57^ which are also the two forms of *AhR* that are suspected in producing KF tolerance.^58^ Similar results were found in the present study, where *AhR2* expression increased in the High 400 ng/L PCB126 dose of NBHKF larvae, and no changes were detected in the SCOKF brains after exposure to 40 ng/L PCB126 (Table S16). Additionally, Whitehead et al. (2012) found tolerant KF populations expressed *AhR* gene battery members in a dose dependent manner with PCB126, including glutathione *S*-transferase (*GST*) and forkhead box (*FOX*) Q1 genes.^59^ Forkhead box proteins are transcription factors that regulate the expression of genes in cell growth, proliferation, differentiation, and longevity and are important to embryonic development.^60,61^ The *GST* gene family encodes genes important to detoxication and toxification mechanisms by conjugation of reduced glutathione.^62^ We also found NBHKF control larva had higher baseline expression levels of gstt2 and foxn4; as well as lower baseline expression levels of *foxo6b*, *foxj3*, *foxp1b*, *foxo1a*, *foxp2*, and *foxg1a*, relative to SCOKF control. Interestingly, the present study did not detect *Cyp* genes at a high enough level to test for differences between treatments, which also may be because this study examined only brain tissue.

Usage of the IBM to translate results from laboratory larvae fish behavior into simulated juvenile cohort survival and growth was a novel way for this study to estimate population-relevant effects of sublethal embryo exposure. This approach has only recently been explored in the field of toxicology, and methodologies are still being assessed, such as the inclusion of different levels of uncertainty^22^ or how to apply information from model fish species such as zebrafish to species of interest such as KF^22,63^ and how to translate laboratory end points to real-world end points (e.g.^64^). One difficult goal in toxicology is the expansion of the risk of pollution to the population level, which could be done through IBMs that simulate this risk, resulting in a better understanding of the impacts of pollution. Individual-based models have shown there are additional long-term detrimental sublethal effects from chemical exposure that are not included when estimating risk from lethal concentrations.^65^ However, these models are rarely validated and may require much more complexity to precisely predict impacts. Even so, with the effort to lessen live organismal toxicological testing, IBMs could offer an alternative as they can include uncertainty and offer estimates of risk characterization.^22^

The AOP framework that was the basis of this study facilitated the organization of biological connections, impacts from neurotoxicant exposure, and comparisons between two separate KF populations and two neurotoxicants. The AOPs constructed here allow us to make connections between diverse biological end points such as gene expression (laboratory), behavior (laboratory), and cohort population metrics (simulated). By making these connections, the AOP framework conceptually demonstrates the potential paths of environmental pollutants impacting hierarchical levels of biological organizations that ultimately predict effects on fish populations and fitness. Effects from both MeHg and PCB126 found in this study will allow for appropriate levels of risk to be assigned to sublethal levels of neurotoxicants in our environment because this study reports effects from exposure from three different levels of biological organization: molecular (brain gene expression), organism (larval swimming and feeding behavior), and cohorts (IBM juvenile growth and survival). These results will provide a more diverse and complete understanding of how contaminants affect the response and long-term persistence of fish populations through the use and connection of the many end points collected over an extended length of time that give a broad picture of the sublethal effects of embryo exposure to juvenile growth and survival.

## Supporting Information Available

Additional detailed methods and results descriptions (PDF)
List of model parameters including effect concentration distributions (TXT)
Significantly differentially expressed genes (alpha = 0.05) found in the brains of Atlantic killifish *F. heteroclitus* (TXT)
Significantly altered gene pathways (alpha = 0.05) found in the brains of Atlantic killifish *F. heteroclitus*(TXT)
Significant PCB126 treatment patterns shared by differentially expressed genes and behavior end points in Atlantic killifish *F. heteroclitus*(TXT)
Significant mercury treatment patterns shared by gene pathways and behavior end points in Scorton Creek (SCO) Atlantic killifish (TXT)
