# Supplementary material for: Impacts on Atlantic Killifish from Neurotoxicants: Genes, Behavior, and Population-Relevant Outcomes
Source: Environ Sci Technol. 2024 Sep 17;58(39):17235–46. doi: 10.1021/acs.est.4c04207 (PMC11447911; doi:10.1021/acs.est.4c04207)
Supplement: Supplementary file 1 — es4c04207_si_001.pdf [file es4c04207_si_001.pdf]

Article Title: Impacts on Atlantic killifish from neurotoxicants: genes, behavior, and population relevant outcomes

Authors: Janice L. Albers [1], Lori N. Ivan [1], Bryan W. Clark [2], Diane E. Nacci [2], Rebekah H. Klingler [3], Adam Thrash [4], Juan P. Steibel [1], Natalia Garcia-Reyero Vinas [5], Michael J. Carvan [3] and Cheryl A. Murphy [1,*]

[1] Department of Fisheries and Wildlife, Michigan State University, East Lansing, MI, USA 48824; albersja@msu.edu (J.A.); steibelj@msu.edu (J.S.); Inivan@msu.edu (L.I.); camurphy@msu.edu (C.M.)

[2] U.S. Environmental Protection Agency, Office of Research and Development, Center for Environmental Measurement and Modeling, Atlantic Coastal Environmental Sciences Division, Narragansett, RI, USA 02882; nacci.diane@epa.gov (D.N.); clark.bryan@epa.gov (B.C.)

[3] School of Freshwater Sciences, University of Wisconsin-Milwaukee, Milwaukee, WI, USA 53204; klinglr@uwm.edu (R.K.); carvanmj@uwm.edu (M.C.)

[4] Mississippi State University, Institute for Genomics, Biocomputing and Biotechnology, Starkville, MS, USA 39759; thrash@igbb.msstate.edu (A.T.)

[5] Environmental Laboratory, US Army Engineer Research and Development Center, US Army Corps of Engineers, Vicksburg, MS, USA 39180; Natalia.G.Vinas@erdc.dren.mil (N.G.R.)

* Correspondence: camurphy@msu.edu; Tel.: 517-432-7771 (C.M.)

63 pages
6 Figures
26 Tables

Supplemental

Behavior Assay Methods

After exposure, 7dpf embryos were rinsed in fresh seawater and transferred into 50-ml conical centrifuge tubes full of fresh seawater (< 50 embryos per tube) and shipped overnight to UWM, where they were placed in 12-well plates and maintained at 23° C (1-2 embryos per well with 1ml artificial seawater; Falcon® Corning, NY 12 well plate 85 x 128 mm, 22 mm diameter well). At 10 dpf, embryos were phenotyped microscopically when abnormalities in developmental stage and features were noted[1,2]. At 14 dpf, plates were rocked gently (~120rpm) and seawater added to each well to initiate hatching. Individual larvae were maintained in single wells for all assessments containing 3 mL seawater, incubated at 23° C, fed 24-h hatched *Artemia* ad lib daily, and renewed with seawater on alternate days. Individuals were assessed daily for survival until 23-24 dpf.

During larvae development, multiple behavior assays were conducted to determine if chemical exposure altered important behavioral milestones. Logistical constraints required two separate batches of fish to be produced (fertilized on August-8-2017 from parents on diets for 103 days and August-21-2017 from parents on diets for 115 days) and for some fish to be included in multiple assays. KF were exposed to MeHg as embryos via parental transfer or were exposed through aqueous solution of PCB126 1-7 dpf. Embryos hatched at 14 dpf, assessed with the Visual Motor Response (VMR) assay at 16 dpf (n=144), a random subset contributed brain samples using lethal methods for gene expression at 17 dpf (n=69, 36 of whom had been through the VMR assay), assessed with Locomotion Behavior assay at 17 dpf (n=256, 108 of whom had been through the VMR assay), and feeding abilities were assessed at 23 or 24 dpf (n=192, 84 of

whom had been through the VMR and Locomotion assay and 192 had been through the Locomotion assay; see Table S1 for the total number of fish in each assay and treatment).

VMR Assay

Visual Motor Response (VMR) assays are a common test of fish neurological system function by startling the fish and evaluating their response[3]. VMRs were conducted using the same methodology as Mora-Zamorano et al. (2017), where 16 dpf larvae were tested in a special behavior chamber while in the transparent 12-well microliter plates. The testing chamber isolated the larvae from light and sound, as described in three previous studies[4–6] and provided adequate light and video surveillance to view all individual movement. VMR assays were conducted between the hours of 1200 and 1800 to minimize within day variability[7]. KF larvae were positioned in a dark behavior chamber and acclimated in the dark for 10 minutes (did not use data during this period), after which they underwent two cycles of alternating 10 min light and dark periods for a total of 50 min. This resulted in larvae used in the VMR analysis experiencing two startles each from dark to light and from light to dark and 4-10 minute periods differing light conditions: two dark and two light. Light levels during the light periods were set to 69 lx based on the work by MacPhail et al. (2009; Fisher Scientific Traceable Dual-Range Light Meter, Pittsburgh, PA).

Spontaneous movement of larvae was constantly recorded at a rate of 30 frames per sec and tracked using DanioVision© system version 8.0 (Noldus Information Technology, Leesburg, VA). Settings for tracking did not include smoothing of track. The minimal distance before movement was recorded was set to 0.2 mm, at which time the direct distance between the two points was calculated. Tracking errors were corrected by plotting all x, y coordinates and

locating and correcting occurrences where the track indicated movement but the fish did not move or track was outside the boundary of the dish. Occasionally when Ethovision lost a fish for 1 to 3 frames (4-SCO-MeHg, 3-NBH-Ctrl, 3-NBH-PCB), the equidistant point/s between the previous and next location were calculated and used as locations.

Similar to Albers et al. (2022), this study used the censored fish locations to define individual larvae activity at each frame within each period. Speed at each frame was calculated as mm per sec and distance traveled in mm. Swimming was defined as larval movement that was at least 6 mm/sec or 0.2 mm per frame (i.e. magnitude of velocity at larvae center) and lasted longer than 5 frames (0.166 sec). Whereas the resting behavior occurred during frames where movement was less than 1 mm/sec or if greater than 1 mm/sec, lasted less than 5 frames. Where resting behavior was defined, speed and distance for those frames were changed to zero. In addition, the turning angle associated with each frame of swimming was calculated using the difference between the four-quadrant inverse tangent of the two trajectories. Where the first trajectory was constructed from the first two locations in the sequence, and the second trajectory from the second two locations in the sequence. This results in a turning angle that ranges from -3.14 to 3.14, where zero is straight ahead movement, negative values indicate right turns and positive values indicate left turns. Swimming bout characteristics (i.e. time between rest periods) were summarized using multiple metrics: number of bouts per second; the mean duration, speed, and turning angle (See Table S3 for definitions). The overall larval behavior during each period in the assay was also summarized using multiple overall summary metrics: total distance traveled, total time swimming, overall average step length and variation, overall turning angle and variation.

The fish larvae responded to the visual startle from the light change as is typical of previous startle responses[3]. Consequently, two behavior endpoints were calculated specifically to determine how larvae responding to the visual startle of the light turning off and on. To determine the magnitude of the response to the visual startle, we determined the frame where the maximum speed was traveled within 5 seconds after the startle. Then the difference between this maximum speed and the speed at the time of the startle was calculated to define the magnitude of the startle response. Startle response time was calculated as the difference in time between the startle and the frame where the maximum speed was traveled.

Locomotion Assay

Typically, KF larvae initiate swimming soon after hatching[9]. The focus of this study was to assess larvae behavior at the point that larvae were independent and actively swimming. Consequently, the locomotion assay was conducted when KF larvae were 17 dpf (3 dph, 6.8 ± 0.67 mm in length, n=180), where each 12-well plate was transferred to the behavior testing chamber. Since previous locomotion assays indicated some neurotoxicants impact larvae only during light periods[4], light levels were constant during the entire assay and set to 69 lx[7]. Assays were conducted during the afternoon between 1200 and 1730 hrs. After an acclimation period of 5 min, spontaneous movement of larvae were tracked every 30th of a second using DanioVision© software 8.0 with the same settings described for the VMR assay. Additionally, DanioVision© lost track of one fish for more than 300 frames, so this fish was not included in the analysis (treatment SCO-PCB).

Using the censored fish locations, the same activity endpoints used the VMR assay were calculated: average swimming bout speed, duration, frequency, turning angle (Table S3).

Additional behavior metrics that summarized other behaviors over the entire assay were also calculated: total distance traveled and swimming time, average step length and turning angle with their respective variations.

Using the same methods as Albers et al. (2022), a Hidden Markov Chain Model (HMM) was constructed for each fish in the locomotion assay (all fish swam at least once) to describe the different behavioral states and used them as additional behavior endpoints to determine effects from chemical exposure. A brief description of the method follows. For each larva and video frame, the step length and turning angle during the assay were used to construct multiple larval specific HMMs using the R package moveHMM[10,11]. Multiple behavior state models were examined that contained three possible swimming states: slow, medium, and fast swimming states where s1 HMMs contained only one behavior state, s2 HMMs contained any two behavior states, and s3 HMMs contained all three behavior states. The best fit HMM for each larvae was determined from a suite of ten potential HMM models, differing in the number of behavior states and initial starting values for each state (see Albers et al. 2022 Table S2 for model description and initial values).

Once all 10 of the possible HMMs were completed, a hierarchical selection for the best fitting model was conducted, essentially using successfully converged models with the lowest AIC. Even though the initial state values were set up in increasing step length means, the resulting best fit HMM state parameter estimates did not always have increasing step length for each additional behavior. This is probably due to the final HMM behavior state being defined by not only the step length but also turning angle characteristics. To make sure the behavior state comparisons were comparing similar states with the same name, the states were reordered and renamed in order to compare between larvae. First, states were reordered using the mean step

length to describe them as slow, medium and faster swimming behavior states (i.e. changed the state name). Next a Linear Discriminant Model was constructed using the lda function in the MASS package[12] and cross validation to compare between models using the s3 models as a reference. LDA prediction accuracy for all models (s1, s2, and s3) was measured using cross validation where a random draw of 80% of the data was used to construct a model and then calculated prediction accuracy of the remaining 20% of the data. This was done 50 times for each treatment group of data to determine overall accuracy (98 ± 0.02 %) and within state accuracy (slow state = 99 ± 0.01 %, medium state = 99 ± 0.01 %, and fast state = 95 ± 0.05 %; Table S4).

When treatment level tests were conducted on slow, medium and fast states, this comparison was only conducted with fish that performed those states making the number of larvae used for the model (see Treatment Testing section below) different for each comparison (Table S1).

Feeding Assay

Typically, KF larvae initiate feeding at 17 dpf[13]. This study focused on assessing larvae behavior at the point that larvae were independent and feeding. Consequently, feeding ability in KF was assessed when they were 23 or 24 dpf (9 or 10 dph; 10.6 ± 0.82 mm). Larva were transferred from the 22 mm diameter wells to 54 mm diameter petri dishes at 22 dpf (60 mm petri dish). Feeding of *Artemia* continued morning and evening until ~24hr prior to the assay, so fish would be in a hungry state for the test. Similar to locomotion assays, feeding assays were conducted over a two-day period between 1300 and 1920 hrs at a light level of 69 lx. Feeding assays were conducted in the same behavior chamber as the locomotion assay, when after 5

minutes of acclimation, recording started and ~15 (range 13-19) live *Artemia* were added to the dish. The test ended when 5 minutes had elapsed from when the *Artemia* were added to the dish.

Feeding bouts consisted of multiple presentations; the characteristic curved body posture, continuously swimming straight or at rest by just opening their mouths. For each of these presentations, the distance between the middle of the larva's mouth and *Artemia* was measured at the time the larva orientated toward the *Artemia*, with their either eyes or body. This distance was termed predator reactive distance and was measured using ImageJ (version 1.51j8). For each capture attempt toward an *Artemia*, we recorded whether the larva successfully captured the *Artemia* and the time it took the larva to handle and consume the *Artemia*. Typically after a catching an *Artemia*, the larva sat or drifted momentarily and did not swim while it was consuming the prey. Handling time was defined as the time between prey capture and when the larva resumed normal swimming activity. Additionally, three consumption metrics were calculated: capture proportion defined as the number of captures divided by the total number of *Artemia* added to the dish, miss proportion defined as the number of feeding capture attempts that missed the *Artemia* divided by the total number of successful and unsuccessful capture attempts, and capture attempt ratio defined as the total number of feeding capture attempts (successful and unsuccessful) divided by the total number of *Artemia* added to the dish. When two *Artemia* were consumed during one feeding capture attempt, the consumption of both *Artemia* were assigned the same measurements.

Bayesian Model Analysis

For each behavioral endpoint (Table S3), we conducted a series of preliminary and final tests to determine whether there were differences between chemical dose treatments. The three different behavior assays and the number of behavior responses we measured were Feeding-5, Visual Motor Response (VMR) - 58, Locomotion - 30. Behavior responses that were not already normally distributed, we attempted to normalize using the boxcox function in the R MASS package[12] (Table S5). Using a basic model containing only the treatment factor, behavior endpoints were transformed using the maximum lambda parameter for the exponential transformation suggested by the boxcox function in the R MASS package. Below we describe the five different models that were used on the 93 behavior responses to determine differences between treatments, (see Table S5 for final transformation and model used for each behavior endpoint). Fitting multiple model types was necessary due to the various behavior endpoints having distinctively different distributions such as a proportional, normal, or a skewed response that even Box Cox transformations were not successful in normalizing.

Model Description

The Bayesian models used in locomotion and VMR behavior response models consisted of one main effect (treatment with 5 levels), covariate variable time of assay and a random batch effect because assays were ran in batches of 24-well dishes. The Bayesian model used for a locomotion and VMR behavior responses was

$$Locomotion\ or\ VMR\ Behavior\ Endpoint_{ijkl} = \alpha + \beta_j * treatment_j + \delta_k * assay\ time_{k(i)} + \omega_l * batch_{l(i)} + \varepsilon_{ijkl}$$

where $Locomotion\ or\ VMR\ Behavior\ Endpoint_{ijkl}$ is the behavioral response metric on the ith individual, j^{th} treatment, k^{th} assay time and l^{th} batch; α is the intercept, β_j is the treatment coefficient with a $Normal\left(0,\sigma_{\beta}^{2}\right)$ distribution, δ is the assay time coefficient with a $Normal\left(0,\sigma_{\delta}^{2}\right)$ distribution, ω_l is the batch coefficient, and ε_{ijkl} is the residual error. Treatment and batch are indicator variables containing 1 if the observation belongs to the corresponding factor category and 0 otherwise. Prior distributions for these two components are described in Table S6. Additionally, priors were needed for the α, treatment and assay time effects. In all models, we used non-informative, flat priors. For α, treatment, and assay time we assumed a normal distribution with a mean of 0 and standard deviation of at least 1.0×10^4 (i.e. precision of 1.0×10^{-4}). OpenBUGS model code for these models is shown in Tables S7, S8 and S9.

Two other Bayesian models were used to model the five feeding behavior responses that did not contain a batch effect since feeding assays were conducted one fish at a time. Additionally, days post fertilization (dpf) was included as a covariate since larvae were either 23 or 24 dpf. Lastly, these models did include intercept, treatment and assay time as described for the locomotion and VMR behavior models.

1) Normal response model

$$Feeding\ Behavior\ Endpoint_{ijkl} = \alpha + \beta_j * treatment_j + \delta * assay\ time_{k(i)} + \omega * dpf_{l(i)} + \varepsilon_{ijkl}$$

where $Feeding\ Behavior\ Endpoint_{ijkl}$ is the prey handling time, lunge ratio or reaction distance (Table S5) on the ith individual, j^{th} treatment, k^{th} assay time and l^{th} dpf; α, β_j and, δ and their priors where described before, and ω is the dpf coefficient also with a non-informative

normal prior assuming a normal distribution with a mean of 0 and standard deviation of at least 1.0 x 10^4 (i.e. precision of 1.0 x 10^{-4}). Lastly, the residual error followed a normal distribution $\varepsilon \sim Normal(0, \sigma_\varepsilon^2)$ with variance $\frac{1}{\sigma_\varepsilon^2} = \tau_j \sim I - Gamma(0.0001,\ 0.0001)$. OpenBUGS code is presented in Table S10.

2) Binomial response model

$$Feeding\ Behavior\ Endpoint_{ijkl} \sim Binomial(p_{ijkl}, N_{ijkl})$$

$$logit(p_{ijkl}) = \beta_j * treatment_{j(i)} + \delta * assay\ time_{k(i)} + \omega * dpf_{l(i)} + \varepsilon_{ijkl}$$

where $Feeding\ Behavior\ Endpoint_{ijkl}$ is the prey capture probability or prey miss proportion (Table S8) on the ith individual, j^{th} treatment, k^{th} assay time and l^{th} dpf and N_{ijkl} is the number of trials and p_{ijkl} is the probability of success distributed on a logit scale. The priors for β_j, δ and ω where described before. Lastly, the residual error followed a normal distribution $\varepsilon \sim Normal(0, \sigma_\varepsilon^2)$ with variance $\frac{1}{\sigma_\varepsilon^2} = \tau_j \sim I - Gamma(0.01,\ 0.01)$. OpenBUGS code is presented in Table S11.

Model Fitting and Convergence Diagnostics

Bayesian models were constructed using OpenBUGS version 3.2.3 rev 1012 [14], R version 3.6.0 [11] and packages R2OpenBUGS version 3.2 [15] and coda version 0.19-2 [16]. We fit the basic model using three chains, each with a minimum of 10000 iterations, 1000 burn in, and 1 thin, and monitored a subsample of parameters for convergence: treatment effects, overall mean, residuals, variance(s), precision parameter(s), and degree of freedom parameter(s). Then we performed preliminary multiple MCMC chain convergence diagnostics using Trace plots. If

model did not converge, we increased either the number of iterations, burn in, or thin. Once the preliminary model trace plots were not showing any obvious convergence problems, further MCMC diagnostics were applied using a suite of tools to determine adequate MCMC chain length, model convergence and fit. 1) Autocorrelation plots indicated the level of thinning required to remove any autocorrelation. 2) Gelman-Rubin-Brooks shrink factor plots indicated the adequate number of iterations needed for burn in. 3) Raftery and Lewis's diagnostic tables were used to determine the number of additional iterations needed for accurate parameter estimation (default values of $q = 0.025$, $r = \pm 0.005$ and $s = 0.95$). 4) Finally, model goodness-of-fit was evaluated using residual diagnostics. When alternative models were to be compared, the model with the best posterior predictive distributions of residuals and replicated observations was retained.

Once a best-fitted model had been determined, we re-fit the model with the appropriate settings and monitored a slightly different suite of parameters: overall mean; population level treatment effects; variance and precision parameters; tail area probabilities of observing a difference; degrees of freedom; individual level predicted means, etc. With the model output and iteration levels we also determined effective sample size (effectiveSize function in coda R package), posterior distributions of parameters, and calculated a one-sided tail area probabilities (Bayesian P-values) from the two sided difference of parameter distributions. The summary output of this last model fit is presented in the results section of the paper and all relevant parameter posterior distributions can be found in Table S13.

Behavior Treatment Testing

All behavioral endpoints were examined for treatment differences using Bayesian statistical methods (see Supplemental section for additional details). Bayesian models for locomotion behavior responses consisted of one main effect (treatment with 5 levels), covariate variable (time of test and/or dpf) and a random batch effect since assays were ran in batches of 12-well dishes. Bayesian models for feeding behavior were the same except no random batch was included since each assay was conducted with one larva. Response variables and residuals were examined for normality using density distributions and Box Cox transformation were applied where needed in all non-negative response variables using the boxcox function in the MASS package (Table S5[212]). All responses that were normally distributed either with or without a transformation were predicted using a normal distribution model, responses that were severely right skewed were predicted using a t distribution model where degrees of freedom (df) was estimated with dunif (3, 30), and responses that were proportional were fit with a logistic distribution model (Tables S6, S7, S8 and S9, S10, respectively). Priors were set to be non-informative and all models were ran with three chains (see supplemental material for detailed methods; Table S6). To facilitate future use of parameter estimates, this study generated both overall population and individual level parameter estimates (Table S12). Lastly, a Chi-Square test from the R stats package[11] was used to determine whether the proportion of s1, s2 and s3 HMMs selected were different between treatments.

Brain Gene Expression

Brain collection

Brain collection was performed essentially as described by Vargas et al. (2011) on 17 dpf MeHg and PCB126 exposed larvae. A random subset of larvae were removed after the VMR

assay to contribute brain samples for gene expression at 17 dpf (n=69, 36 of whom had been through the VMR assay and 33 had not). Larvae were gently transferred to a 60 mm petri dish and 4°C embryo medium was quickly added to provide anesthesia. Five larvae were transferred to a new petri dish, water was removed, and individuals were immobilized in a drop of 2% low melting point agarose made with artificial cerebral spinal fluid (aCSF; 131 mM NaCl, 2 mM KCl, 1.25 mM KH_2PO_4, 2 mM $MgSO_4$, 10 mM glucose, 2.5 mM $CaCl_2$, 20 mM $NaHCO_3$). A dissection pin was used to mount the larvae in dorsal/ventral recumbency, just under the surface of the agarose. Artificial cerebral spinal fluid was added and dishes were placed on ice. Intact brains were removed using dissection pins, transferred individually in 5µl aCSF to 1.5 ml microcentrifuge tubes, then frozen in liquid nitrogen prior to storage at -80°C.

Brain Gene Analysis

Genomic analysis was conducted at Mississippi State University, Institute for Genomics, Biocomputing and Biotechnology. Total RNA was isolated from 6 embryos' brains per treatment from individual 17 dpf embryos using the Qiagen RNeasy® Micro Kit (Germantown, MD, USA) following the Purification of Total RNA from Animal and Human Tissues protocol in the RNeasy® Micro Handbook with slight modifications. The modification included homogenization of brain tissue in 350 µL of RLT buffer using a pellet pestle and elution of Total RNA using 15 µL of RNase-free water. RNA quality was assayed using the Agilent High Sensitivity RNA ScreenTape System (Waldbronn, Germany) for the Agilent 2200 TapeStation (Palo Alto, CA, USA), and RNA was quantified using the NanoDrop 2000 (ThermoFisher Scientific, Waltham, MA).

The raw reads from 36 KF samples (6 groups with 6 reps) were mapped and quantified using salmon[18] (v1.3.0) against the reference transcriptome (NCBI *Fundulus heteroclitus* annotation release 102; assembly MU-UCD_Fhet_4.1). Tximport[19] (v1.16.1) was used to import transcript-level estimates from salmon summarize this data to the gene level. These genes were filtered such that only genes with an average log Counts per Million > 1 across all samples were retained for differential expression analysis. EdgeR (v3.30.3) was used to determine differentially expressed genes (DEGs). OrthoFinder (v2.5.4) was used to find orthologous genes in *D. rerio.* The GO.db R package [20] (v3.12.1) was used to look up GO terms from GO IDs, and the KEGGREST R package [21] (v1.30.1) was used to look up KEGG pathway names. The GAGE R package[22] (v2.40.0) was used to perform gene-set enrichment analysis using *D. rerio* GO gene-sets, KEGG gene-sets and the *D. rerio* orthologs of genes that passed the filter. Significant trends were determined using an alpha of 0.05 [false discovery rate (FDR) and q-value]. Brain gene expression data for each embryo in each treatment can be found at www.ncbi.nlm.nih.gov/geo.

Individual Based Model

The model used in this study is described in Ivan et al. (In Review) with a few changes. First, the calibration in this study was unique to KF and did not include any other species. Second, the model in this study did not contain uncertainty as described in Ivan et al. (In Review). Lastly, this study added an additional time period of summer since KF have an extended spawning season and we wanted to investigate the possibility that seasonal changes in predation may occur.

The IBM tracked 2500 individual larvae (based on wild densities) from hatch to juvenile transition, defined at 24 mm [23] or until 100 days, whichever occurred first (Figure S2). Daily, individuals forage, grow and experience mortality. Killifish forage on two types of prey. Foraging consists of prey encounters, handling time, capture success and consumption of nauplii and/or copepods. Swimming speed, handling time, larvae reactive distance and capture success all determine how many prey an individual KF larval consumes. KF then grow ($G_{j,d}$ in g/d) as

$$G_{j,d} = C_{j,d} - R_{j,d} - F_{j,d} - U_{j,d} - SDA_{j,d}$$

where $C_{j,d}$ (g/d) is the consumption of prey by larval fish j, $R_{j,d}$ is respiration (g/d), $F_{j,d}$ is egestion (g/d), $U_{j,d}$ is the excretion (g/d) and $SDA_{j,d}$ (g/d) is the specific dynamic action. Consumption is determined via the foraging but capped at $Cmax_{j,d}$ (g/d) as determined from the Wisconsin Bioenergetic equations[24]. Finally, KF are monitored for starvation and predation mortality. Predators of KF are adult KF and their predation rates are temperature dependent[24]. Fish that die are removed from the daily loop, as are fish that reach 24mm. Output variables of interest are 1) the number of survivors (fry that reach the exit length within the 100 days) and 2) the mean growth rate (mm/d) of survivors.

Sublethal effects of MeHg and PCB126 were incorporated into the model via multipliers derived from the Bayesian individual level predicted treatment posterior distributions (Table S13). The individual level posterior distributions were used to create 10,000 random values from a truncated normal distribution. If the posterior distribution was from a transformed behavior endpoint, then these random values were back transformed. From these random values, the multiplier distributions were generated (S12). Multipliers were placed on larval swimming speed from the locomotion assay; larval capture success of zooplankton, larval handling time of zooplankton, and larval reactive distance to zooplankton from the feeding assay. At the start of

each simulation (replication), each model individual j was assigned a multiplier for each of the above four variables. For each simulated KF (j), a swimming speed multiplier (SM_j) was generated as

$$SM_j = TD_j/MD$$

where TD_j is the average speed (mm/s) by fish j and MD is the treatment mean average speed (mm/s). Multipliers for handling time ($HM_j = TH_j/MH$), capture success ($CM_j = TC_j/MC$) and reactive distance ($RM_j = TR_j/MR$) were calculated for each experimental fish j as using the same procedure. Finally, the amount of time a fish was active was determined by the proportion of time fish were active in the locomotion assay. Proportions were derived from the posterior distributions for each scenario. If necessary, back-transformations were performed prior to the multiplier calculation. Lastly, the proportion of time a KF was actively searching for food or encountering a predator was scaled to the percent of time active larvae were in the locomotion assay by randomly assigning a time scaler to each fish at the beginning of the simulation (i.e. multiply 12 hours by percent of time active in assay).

The model was calibrated using SCO KF such that growth rates were set to be approximately 0.3mm/d (unpublished). To determine if differences occurred between which season the adult fish spawn, we ran simulations for spring and summer runs. For spring runs beginning on Julian day 110, the first fish reached 24mm around day 53 with several individuals still growing but under the size of 24mm at the end of the model run (Figure S4). For the summer runs (Julian day 230), the first fish to reach 24mm at the end of the model run was on day 48 with few fish remaining in the simulation at the end of the model run (Figure S4).

Results

All larvae within each chemical/year/treatment group were successfully fitted with a HMM (Table S1). The number of larvae that consisted of one, two, or three behavior states exhibited a consistent pattern within each treatment, where a one state behavior model was never the best, three fish exhibited a two state model, and the rest of the fish were best fit using a three state behavior model (253 fish; Figure S3).

Behavior/Gene Expression

This study focused on finding behaviors that reacted in a similar pattern to either MeHg or PCB126 treatments. In addition, we also tested individual genes and whether they responded in a similar pattern as any of the behavior endpoints. These results are reported in Tables 2 and S18 to S24.

References

(1) Clark, B. W.; Matson, C. W.; Jung, D.; Di Giulio, R. T. AHR2 Mediates Cardiac Teratogenesis of Polycyclic Aromatic Hydrocarbons and PCB-126 in Atlantic Killifish (Fundulus Heteroclitus). *Aquat. Toxicol.* **2010**, *99* (2), 232–240. https://doi.org/10.1016/j.aquatox.2010.05.004.

(2) Whitehead, A.; Triant, D. A.; Champlin, D.; Nacci, D. Comparative Transcriptomics Implicates Mechanisms of Evolved Pollution Tolerance in a Killifish Population. *Mol. Ecol.* **2010**, *19* (23), 5186–5203. https://doi.org/10.1111/j.1365-294X.2010.04829.x.

(3) Emran, F.; Rihel, J.; Dowling, J. E. A Behavioral Assay to Measure Responsiveness of Zebrafish to Changes in Light Intensities. *J. Vis. Exp.* **2008**, No. 20, 6. https://doi.org/10.3791/923.

(4) Mora-Zamorano, F. X.; Klingler, R.; Basu, N.; Head, J.; Murphy, C. A.; Binkowski, F. P.; Larson, J. K.; Carvan, M. J. Developmental Methylmercury Exposure Affects Swimming Behavior and Foraging Efficiency of Yellow Perch (Perca Flavescens) Larvae. *ACS Omega* **2017**, *2* (8), 4870–4877. https://doi.org/10.1021/acsomega.7b00227.

(5) Mora-Zamorano, F. X.; Klingler, R. H.; Murphy, C. A.; Basu, N.; Head, J.; Carvan, M. J. Parental Whole Life Cycle Exposure to Dietary Methylmercury in Zebrafish (Danio Rerio) Affects the Behavior of Offspring. *Environ. Sci. Technol.* **2016**, *50* (9), 4808–4816. https://doi.org/10.1021/acs.est.6b00223.

(6) Mora-Zamorano, F. X.; Svoboda, K. R.; Carvan, M. J. The Nicotine-Evoked Locomotor Response: A Behavioral Paradigm for Toxicity Screening in Zebrafish (Danio Rerio)

Embryos and Eleutheroembryos Exposed to Methylmercury. *PLoS One* **2016**, *11* (4), e0154570. https://doi.org/10.1371/journal.pone.0154570.

(7) MacPhail, R. C.; Brooks, J.; Hunter, D. L.; Padnos, B.; Irons, T. D.; Padilla, S. Locomotion in Larval Zebrafish: Influence of Time of Day, Lighting and Ethanol. *Neurotoxicology* **2009**, *30* (1), 52–58. https://doi.org/10.1016/J.NEURO.2008.09.011.

(8) Albers, J. L.; Steibel, J. P.; Klingler, R. H.; Ivan, L. N.; Garcia-Reyero, N.; Carvan, M. J.; Murphy, C. A. Altered Larval Yellow Perch Swimming Behavior Due to Methylmercury and PCB126 Detected Using Hidden Markov Chain Models. *Environ. Sci. Technol.* **2022**, *56* (6), 3514–3523. https://doi.org/10.1021/acs.est.1c07505.

(9) Weis, J. S.; Weis, P. Swimming Performance and Predator Avoidance by Mummichog (Fundulus Heteroclitus) Larvae after Embryonic or Larval Exposure to Methylmercury. *Can. J. Fish. Aquat. Sci.* **1995**, *52* (10), 2168–2173. https://doi.org/10.1139/f95-809.

(10) Michelot, T.; Langrock, R.; Patterson, T. A. MoveHMM: An <scp>R</Scp> Package for the Statistical Modelling of Animal Movement Data Using Hidden Markov Models. *Methods Ecol. Evol.* **2016**, *7* (11), 1308–1315. https://doi.org/10.1111/2041-210X.12578.

(11) R Core Team. *R: A Language and Environment for Statistical Computing*; R version 4.0.4, x86_64-w64-mingw32/x64 (64-bit): Vienna, Austria, 2019.

(12) Venables, W. N.; Ripley, B. D. *Modern Applied Statistics with S*, Fourth.; Springer New York, 2002.

(13) Weis, J. S.; Weis, P. Effects of Embryonic Exposure to Methylmercury on Larval Prey-Capture Ability in the Mummichog, Fundulus Heteroclitus. *Environ. Toxicol. Chem.* **1995**, *14* (1), 153–156. https://doi.org/10.1002/etc.5620140117.

(14) Lunn, D.; Spiegelhalter, D.; Thomas, A.; Best, N. The BUGS Project: Evolution, Critique and Future Directions. *Stat. Med.* **2009**, *28* (25), 3049–3067. https://doi.org/10.1002/sim.3680.

(15) Sturtz, S.; Ligges, U.; Gelman, A. R2WinBUGS : A Package for Running WinBUGS from R. *J. Stat. Softw.* **2005**, *12* (3). https://doi.org/10.18637/jss.v012.i03.

(16) Plummer, M.; Best, N.; Cowles, K.; Vines, K. CODA: Convergence Diagnosis and Output Analysis for MCMC. *R News* **2005**, *6*, 7–11.

(17) Vargas, R.; Jóhannesdóttir, I. þ.; Sigurgeirsson, B.; Þorsteinsson, H.; Karlsson, K. Æ. The Zebrafish Brain in Research and Teaching: A Simple in Vivo and in Vitro Model for the Study of Spontaneous Neural Activity. *Adv. Physiol. Educ.* **2011**, *35* (2), 188–196. https://doi.org/10.1152/advan.00099.2010.

(18) Patro, R.; Duggal, G.; Love, M. I.; Irizarry, R. A.; Kingsford, C. Salmon Provides Fast and Bias-Aware Quantification of Transcript Expression. *Nat. Methods* **2017**, *14* (4), 417–419. https://doi.org/10.1038/nmeth.4197.

(19) Soneson, C.; Love, M. I.; Robinson, M. D. Differential Analyses for RNA-Seq: Transcript-Level Estimates Improve Gene-Level Inferences. *F1000Research* **2015**, *4*, 1521. https://doi.org/10.12688/f1000research.7563.1.

(20) Carlson, M. GO.Db: A Set of Annotation Maps Describing the Entire Gene Ontology R Package Verson 3.12.1. 2019.

(21) Tenenbaum, D. KEGGREST: Client-Side REST Access to KEGG R Package Version 1.30.1. 2015.

(22) Luo, W.; Friedman, M. S.; Shedden, K.; Hankenson, K. D.; Woolf, P. J. GAGE: Generally Applicable Gene Set Enrichment for Pathway Analysis. *BMC Bioinformatics* **2009**, *10* (1), 161. https://doi.org/10.1186/1471-2105-10-161.

(23) Abraham, B. J. *Species Profiles Life Histories and Environmental Requirements of Coastal Fishes and Invertebrates Mid-Atlantic USA Mummichog and Striped Killifish*; U S Fish and Wildlife Service Biological Report 8211, 223 p., 1985.

(24) Deslauriers, D.; Chipps, S. R. S. R.; Breck, J. E. J. E.; Rice, J. A. J. A.; Madenjian, C. P. C. P. Fish Bioenergetics 4.0: An R-Based Modeling Application. *Fisheries* **2017**, *42* (11), 586–596. https://doi.org/10.1080/03632415.2017.1377558.

(25) Smith, K. J.; Taghon, G. L.; Able, K. W. Trophic Linkages in Marshes: Ontogenetic Changes in Diet for Young-of-the-Year Mummichog, Fundulus Heteroclitus. In *Concepts and Controversies in Tidal Marsh Ecology*; Weinstein, M. P., Kreeger, D. A., Eds.; Kluwer Academic Publishers: Dordrecht, 2002; pp 121–237. https://doi.org/10.1007/0-306-47534-0_11.

(26) Marteinsdottir, G.; Able, K. W. Influence of Egg Size on Embryos and Larvae of Fundulus Heteroclitus (L.). *J. Fish Biol.* **1992**, *41*, 883–896.

(27) Kneib, R. T.; Parker, J. H. Notes: Gross Conversion Efficiencies of Mummichog and Spotfin Killifish Larvae from a Georgia Salt Marsh. *Trans. Am. Fish. Soc.* **1991**, *120* (6), 803–809. https://doi.org/10.1577/1548-8659(1991)120<0803:NGCEOM>2.3.CO;2.

(28) Letcher, B. H.; Rice, J. A.; Crowder, L. B.; Rose, K. A. Variability in Survival of Larval Fish: Disentangling Components with a Generalized Individual-Based Model. *Can. J. Fish. Aquat. Sci.* **1996**, *53* (4), 787–801. https://doi.org/10.1139/f95-241.

(29) Fleeger, J. W.; Johnson, D. S.; Galván, K. A.; Deegan, L. A. Top-down and Bottom-up Control of Infauna Varies across the Saltmarsh Landscape. *J. Exp. Mar. Bio. Ecol.* **2008**, *357* (1), 20–34. https://doi.org/10.1016/j.jembe.2007.12.003.

(30) Fulford, R. S.; Rice, J. A.; Miller, T. J.; Binkowski, F. P.; Dettmers, J. M.; Belonger, B. Foraging Selectivity by Larval Yellow Perch (Perca Flavescens): Implications for Understanding Recruitment in Small and Large Lakes. *Can. J. Fish. Aquat. Sci.* **2006**, *63* (1), 28–42. https://doi.org/10.1139/f05-196.

(31) Hartman, K. J.; Brandt, S. B. Predatory Demand and Impact of Striped Bass, Bluefish, and Weakfish in the Chesapeake Bay: Applications of Bioenergetics Models. *Can. J. Fish. Aquat. Sci.* **1995**, *52* (8), 1667–1687. https://doi.org/10.1139/f95-760.

(32) Walton, W. E.; Hairston, N. G.; Wetterer, J. K. Growth-Related Constraints on Diet Selection by Sunfish. *Ecology* **1992**, *73* (2), 429–437. https://doi.org/10.2307/1940750.

(33) Valiela, I.; Wright, J. E.; Teal, J. M.; Volkmann, S. B. Growth, Production and Energy Transformations in the Salt-Marsh Killifish Fundulus Heteroclitus. *Mar. Biol.* **1977**, *40* (2), 135–144. https://doi.org/10.1007/BF00396259.

(34) Madon, S. P.; Williams, G. D.; West, J. M.; Zedler, J. B. The Importance of Marsh Access to Growth of the California Killifish, Fundulus Parvipinnis, Evaluated through Bioenergetics Modeling. *Ecol. Modell.* **2001**, *136* (2–3), 149–165. https://doi.org/10.1016/S0304-3800(00)00416-6.

(35) Cowan, J. H. J.; Houde, E. D.; Rose, K. A. Size-Dependent Vulnerability of Marine Fish Larvae to Predation: An Individual-Based Numerical Experiment. *ICES J. Mar. Sci.* **1996**, *53*, 23–37.

Figure S1. Behavior assays used in this study to collect data on Atlantic killifish larvae for assessment of chemical responses and for inputs into the Individual Based Model.

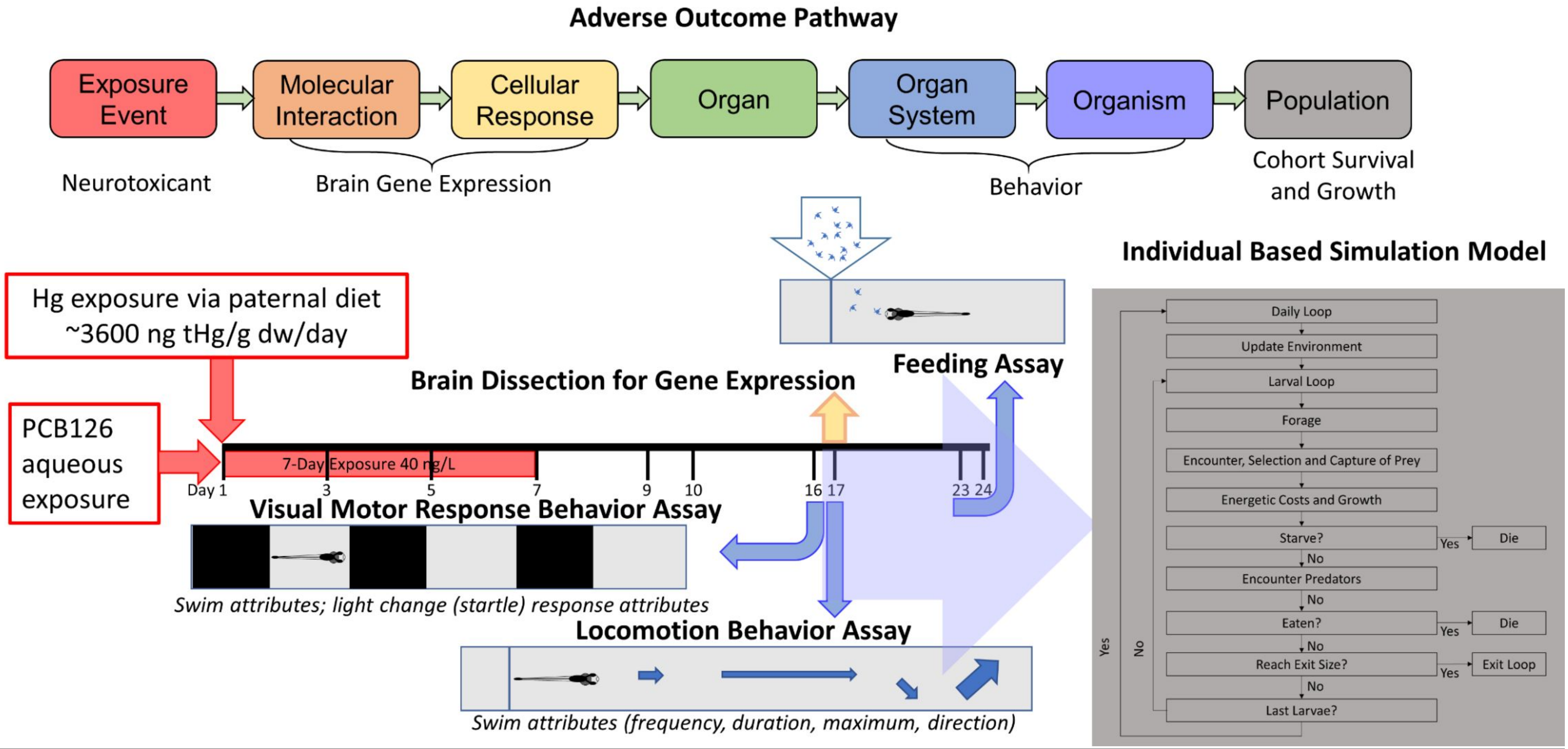

Figure S2. Model flow chart showing daily processes included in the generalized individual-based model to assess contaminant effects on Atlantic killifish larval cohorts.

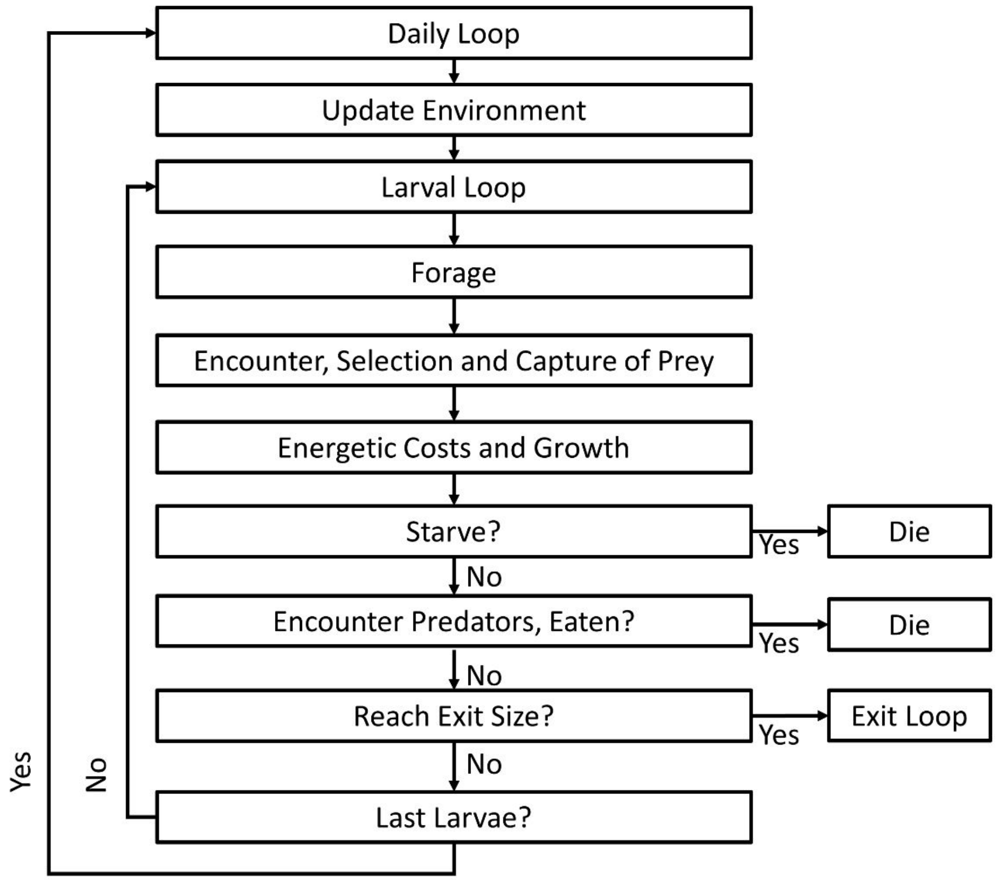

Figure S3. Number of best fit hidden Markov models for Atlantic killifish larvae in the locomotion assay that contained two or three different behavior states.

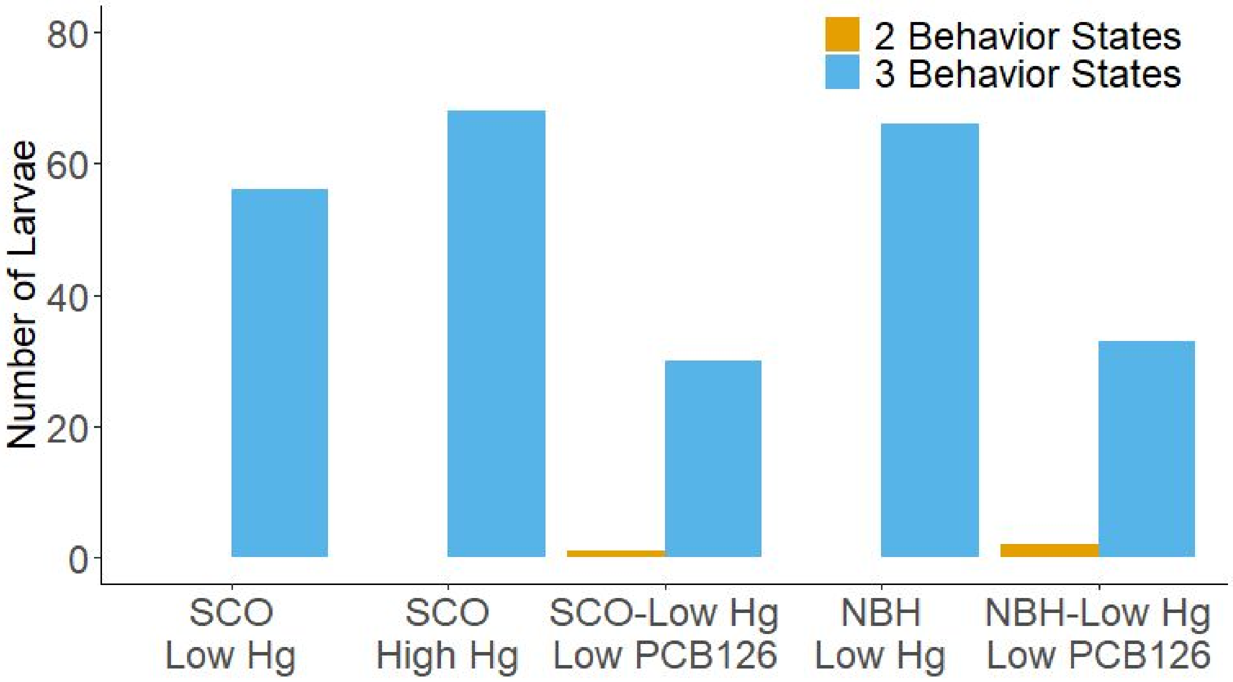

Figure S4. An example of the length (mm) verses simulation day for individual Scorton Creek control fish that were alive at the end of one run of a spring and summer scenario.

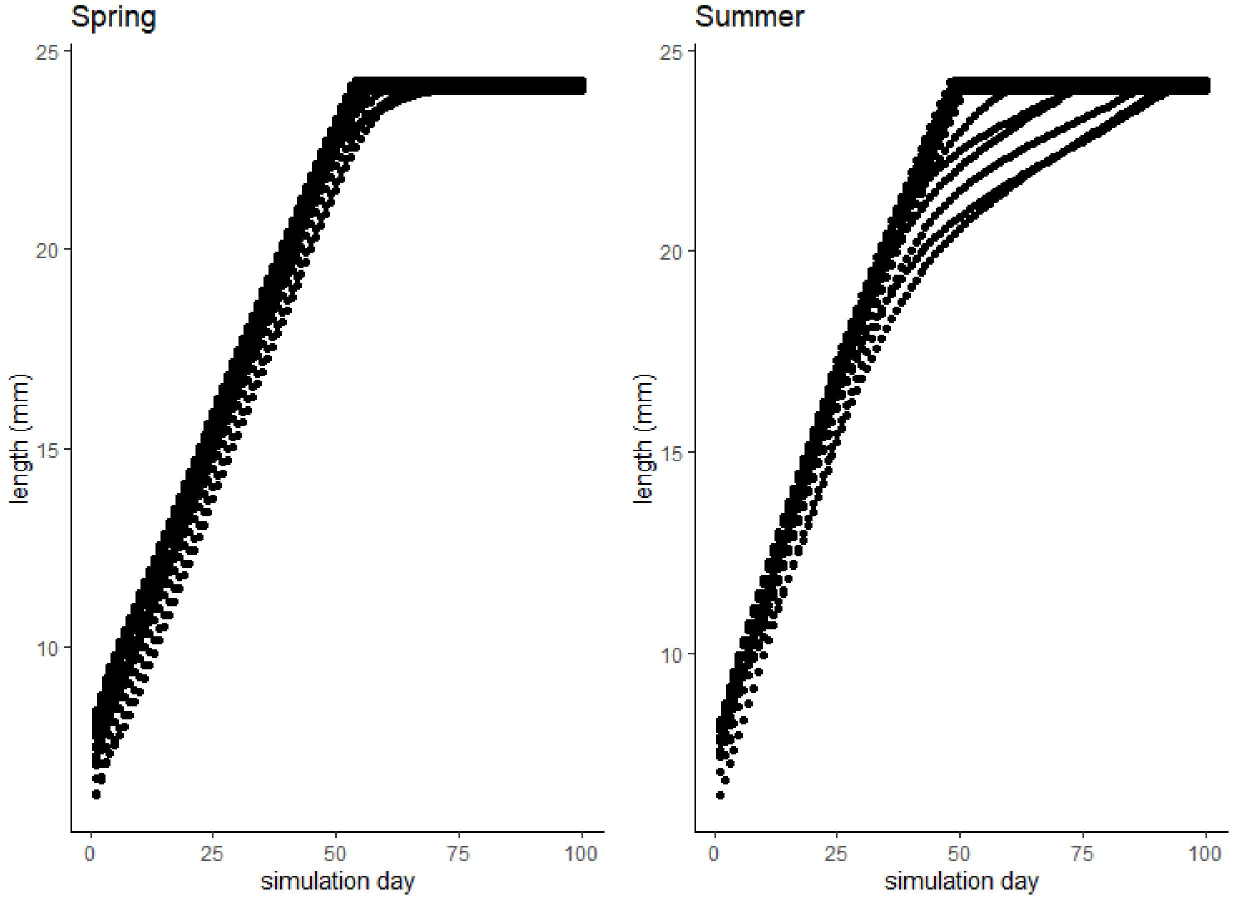

Figure S5. Significant mercury response patterns shared by gene expression and behavior endpoints in Scorton Creek (SCO) Atlantic killifish found in this study. Both the original and opposite behavior endpoint trends are listed. (HMM = Hidden Markov Chain model endpoint, TP = Transition Probability).

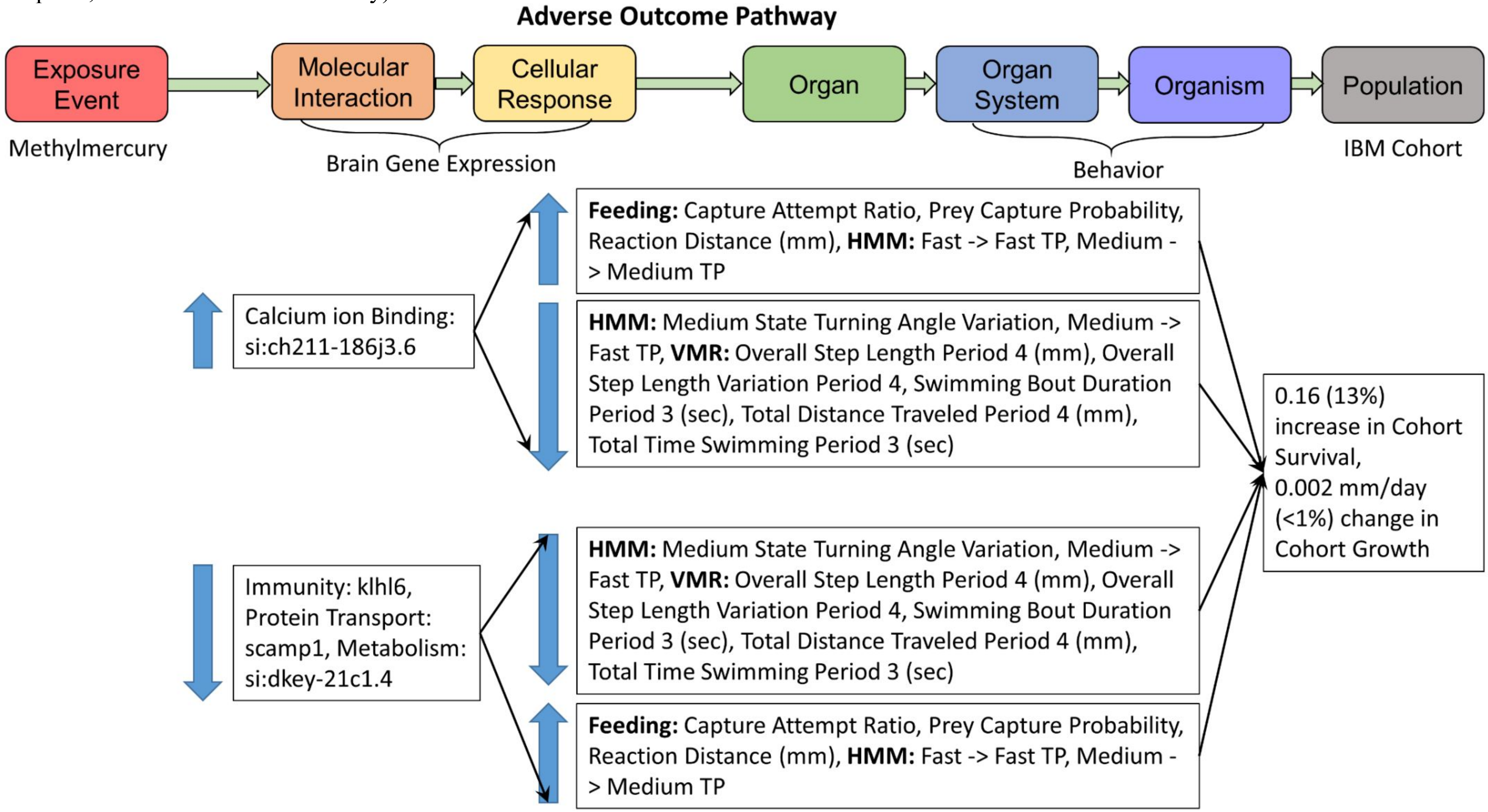

Figure S6. Significant PCB126 treatment patterns shared by gene expression and behavior endpoints in the New Bedford Harbor (NBH) Atlantic killifish found in this study. Both the original and opposite behavior endpoint trends are listed. (HMM = Hidden Markov Chain model endpoint, TP = Transition Probability).

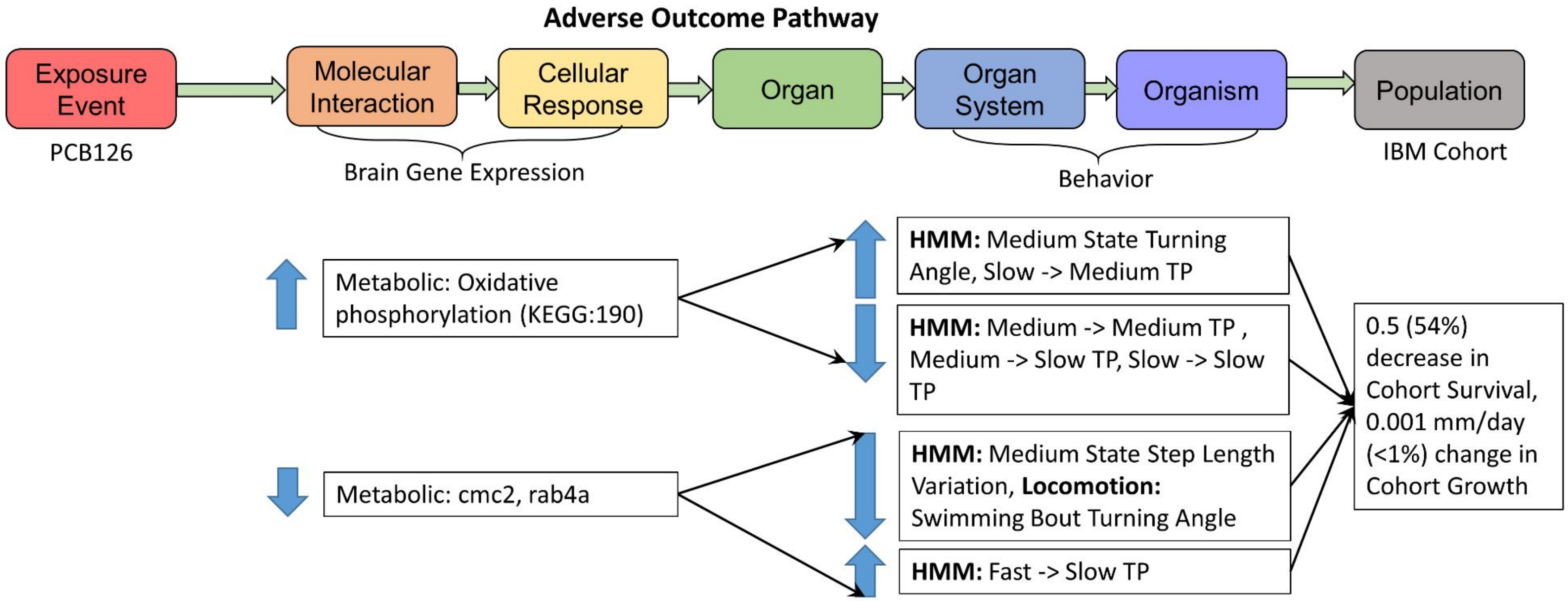

Table S1. Summary of the number of assays and Atlantic killifish larvae (*Fundulus heteroclitus*) used in this study

Groups	SCO-Ctrl	SCO-Hg	SCO-PCB	NBH-Ctrl	NBH-PCB
VMRs					
Number of Assays-Larvae	30	29	28	29	28
Locomotion Assays					
Number of Assays-Larvae	56	68	31	66	35
HMMs					
Number of Larvae Attempted and Fitted a Model	56	68	31	66	35
Feeding Assays					
Number of Assays-Larvae	47	44	23	50	28
Total Length (mm ± SD)	10.78 (0.77)	10.63 (0.60)	9.80 (0.98)	10.81 (0.77)	10.35 (0.80)
Number of Larvae that did not consume Artemia	0	1	0	0	0

Table S2. Embryo treatment groups used in larval behavioral assays, where Atlantic killifish larvae originated from adults from Scorton Creek, MA (SCO) or New Bedford Harbor, MA (NBH). Larvae were fed low mercury (i.e. control) or high mercury (Hg) diets and exposed directly to PCB126 at nominal concentrations of 40 ng/L (Low PCB) or 400 ng/L (High PCB). Endpoints reported include hatching, survival and ratings for phenotypic abnormalities, including those specific to the heart. Lethal treatment groups (PCB126_400) were not used in larval behavior studies (DND = Did not determine, NA = not applicable).

Parents	Parent or Offspring Treatment	Treatment Number	PCB126 ng/g[a]	Mercury ng/g	%Embryo Survival	%Hatch	%Larval Survival	Phenotypic Abnormalities Mean Score	Heart Abnormalities Mean Score
SCO	Control[b]	1	0	9.8 ± 2.49	100	100	90	0	0
	Hg ~3600 ng tHg/g dw/day	2	NA	35.09 ± 17.06	100	87.5	87.5	0.13	0
	PCB126 40 ng/L[b]	3	19	DND	100	100	87.5	0.25	0.63
	PCB126 400 ng/L[b]	N/A	189	DND	100	0	0	4.6	4
NBH	Control[b]	4	0	DND	100	100	100	0	0
	PCB126 40 ng/L[b]	5	19	DND	85.71	85.71	85.71	0	0
	PCB126 400 ng/L[b]	N/A	189	DND	66.67	66.67	55.56	0.86	0.86

[a] Estimated using previous experiments (Nacci et al. 1999)

[b] Also exposed to ~300 ng tHg/g dw/day through salmon-based diet

Table S3. Description of behavior endpoints examined in this study.

Behavior Endpoint	Definition
Feeding Assay	
Prey Capture Probability	The number of artemia captures divided by the total number of artemia added to the assay
Prey Handling Time (sec)	The number of seconds between the prey capture attempt and resuming normal activity, averaged over all feeding capture attempts during the 5 min assay
Capture Attempt Ratio	The total number of prey capture attempts divided by the total number of artemia added to the assay.
Prey Miss Proportion	The number of prey capture attempts that missed the artemia divided by the total number of prey capture attempts during the assay.
Reaction Distance (proportion of body length)	The distance (mm) between the artemia and larva when the larvae first orientates (notices) the artemia divided by the larva total length (mm), averaged over all the feeding capture attempts during the 5 minute assay.
Visual Motor Response Assay	
Startle Magnitude (mm)	Per frame maximum speed within 5 seconds after the startle minus the speed at the time of the startle.
Startle Response time (sec)	Difference in time between the startle and the maximum speed traveled within 5 seconds after the startle
Locomotion and VMR Assay	
Swimming Bouts (per sec)	The number of active swimming bouts per second. Swimming was defined as movement at least 1 mm/s for more than 5 frames (0.166 sec).
Swimming Bout Duration (sec)	Duration of all swimming bouts averaged over the 5 minute period.
Swimming Bout Speed (mm/s)	Per frame swimming speed averaged during a swimming bout; average bout speed averaged over the 5 minute period.
Swimming Bout Turning Angle	Per frame turning angle averaged during a swimming bout; individual average bout turning angle averaged over the 5 minute period. Ranges from -3.14 to 3.14, where negative values indicate right turns and positive values indicate left turns.
Total Distance Traveled (mm)	Total distanced traveled during swimming bouts for the entire 5 minute assay.
Total Time Swimming (sec)	Total time larvae were swimming during 5 minute test.
Overall Step Length (mm)	Per frame distance traveled during a 0.033 second period (one frame to the next) averaged over the entire 5 minute test [i.e. includes zeros when fish moved less than 1 mm/s for more than 5 frames (0.166 sec)].
Overall Step Length Variation	Standard deviation of distance traveled during 0.033 second period (one frame to the next).
Overall Turning Angle	Per frame turning angle averaged over frames when fish were swimming. Ranges from -3.14 to 3.14, where negative values indicate right turns and positive values indicate left turns.
Overall Turning Angle Variation	Standard deviation of per frame turning angle during 0.033 second period (one frame to the next).
HMM Model Parameters	
Step Length (mm)	Per frame distance traveled during a 0.033 sec period (one frame to the next) while the larvae was in each behavior state.

Step Length Variation	Standard deviation of the per frame distance traveled during 0.033 second period (one frame to the next) while in each behavior state.
Turning Angle	Per frame turning angle while in each behavior state. Ranges from -3.14 to 3.14, where negative values indicate right turns and positive values indicate left turns.
Turning Angle Variation	Angle concentration, i.e. kappa parameter in the von Mises distribution while in each behavior state.
Count	Number of frames a behavior state was performed.
Slow -> Slow, Medium -> Slow, Slow -> Medium, Medium -> Medium, Fast -> Slow, Fast -> Medium, Slow -> Fast, Medium -> Fast, Fast -> Fast	Per frame transition probability from state to state (e.g. Medium -> Slow is the probability of a fish transitioning from a medium speed swimming state to a slow swimming state).

Table S4. LDA cross validation results for different HMM behavioral states. N = 50 iterations. SD = standard deviation

Group	Overall Accuracy s3		Slow State		Medium State		Fast State		Number of observations in s3 LDA	Total number of behavior states in s1 and s2	Number of renamed state ID in s1 and s2	Number of larvae with s3	Number of larvae with s2	Number of larvae with s1
	Mean	SD	Mean	SD	Mean	SD	Mean	SD						
NBH-Ctrl	1	0.01	1.00	0.00	1.00	0.00	0.98	0.03	198	0	0	66	0	0
NBH-PCB	1	0.00	1.00	0.00	1.00	0.00	1.00	0.00	108	4	1	36	2	0
SCO-Ctrl	1.00	0.10	1.00	0.01	1.00	0.01	0.99	0.02	168	0	0	56	0	0
SCO-Hg	0.96	0.03	0.98	0.03	1.00	0.00	0.90	0.07	204	0	0	68	0	0
SCO-PCB	0.99	0.02	1.00	0.00	0.98	0.05	0.97	0.06	90	2	0	30	1	0

Table S5. Model summary for each behavioral endpoint.

Parameter	Transformation	Distribution	Number of Larvae	Chains	Iterations	Burn-In	Thin	Sample
Feeding Assay								
Prey Capture Probability	log(y/(1-y))	Logistic	192	3	55000	15000	20	120000
Prey Handling Time	(y+1)^-2.1, y*100	Normal	191	3	30000	10000	10	60000
Capture Attempt Ratio	(y+1)^-1.1, y*100	Normal	192	3	30000	10000	10	60000
Prey Miss Proportion	log(y/(1-y))	Logistic	192	3	35000	10000	10	75000
Reaction Distance (mm)	(y+1)^1.5, y*100	Normal	192	3	30000	10000	10	60000
Locomotion Assay								
Swimming Bouts (per sec)	(y+1)^3.85, y*100	Normal	256	3	165000	45000	20	360000
Swimming Bout Duration (sec)	(y+1)^-3.3, y*100	Normal	256	3	80000	50000	20	90000
Swimming Bout Speed (mm/s)	(y+1)^-1, y*1000	Normal	256	3	175000	35000	20	420000
Swimming Bout Turning Angle	(y+1)^2.59, y*1000	Normal	256	3	620000	50000	15	1710000
Total Distance Traveled (mm)	(y+1)^0.22, y*100	Normal	256	3	210000	25000	20	555000
Total Time Swimming (sec)	(y+1)^0.8	Normal	256	3	805000	35000	20	2310000
Overall Step Length (mm)	(y+1)^-6.7, y*100	Normal	256	3	375000	45000	30	990000
Overall Step Length Variation	(y+1)^-3.7, y*100	Normal	256	3	80000	40000	20	120000
Overall Turning Angle	(y+1)^0.25, y*1000	Normal	256	3	60000	40000	15	60000
Overall Turning Angle Variation	(y+1)^-1.2, y*100	Normal	256	3	90000	40000	20	150000
HMM Model Parameters								
Slow State								
Count	(y+1)^1.41	Normal	256	3	60000	40000	10	60000
Medium State								
Step Length (mm)	(y+1)^-30, y*100000	Normal	255	3	115000	40000	20	225000
Step Length Variation	(y+1)^-30, y*100	Normal	255	3	60000	40000	10	60000
Turning Angle	y*10	Normal	255	3	100000	40000	20	180000
Turning Angle Variation	(y+1)^-0.65, y*100	Normal	255	3	110000	40000	20	210000
Count	(y+1)^0.05, y*100	Normal	255	3	60000	40000	15	60000
Fast State								

Step Length (mm)	(y+1)^-4.3, y*1000	Normal	254	3	150000	40000	20	330000
Step Length Variation	(y+1)^-6.4, y*100	Normal	254	3	60000	40000	15	60000
Turning Angle	y*10	Normal	254	3	80000	60000	15	60000
Turning Angle Variation	(y+1)^0.2, y*100	Normal	254	3	80000	40000	20	120000
Count	NA	Normal	254	3	265000	40000	30	675000
State Transition Probabilities								
Slow -> Slow	asin(sqrt(y)), y*100	Normal	256	3	80000	50000	10	90000
Medium -> Slow	y*100	Normal	255	3	485000	55000	20	1290000
Slow -> Medium	asin(sqrt(y)), y*100	Student's T	255	3	75000	40000	20	105000
Medium -> Medium	y*100	Normal	255	3	125000	50000	20	225000
Fast -> Slow	(y+1)^-71.2, y*100	Student's T	254	3	60000	30000	10	90000
Fast -> Medium	(y+1)^-5.85, y*100	Normal	253	3	110000	50000	20	180000
Slow -> Fast	asin(sqrt(y)), y*100	Normal	254	3	85000	55000	15	90000
Medium -> Fast	(y+1)^-5.1, y*100	Normal	253	3	195000	45000	20	450000
Fast -> Fast	(y+1)^8.6	Normal	254	3	265000	55000	20	630000
VMR Assay								
Period 1, Dark to Light								
Startle Magnitude (mm)	(y+1)^0.18, y*100	Normal	144	3	85000	40000	15	135000
Startle Time (sec)	(y+1)^-0.96, y*100	Normal	144	3	80000	40000	10	120000
Period 2, Light to Dark								
Startle Magnitude (mm)	(y+1)^-0.18, y*1000	Normal	144	3	90000	40000	15	150000
Startle Time (sec)	(y+1)^0.04, y*1000	Normal	144	3	60000	40000	15	60000
Period 3, Dark to Light								
Startle Magnitude (mm)	(y+1)^0.45, y*1000	Normal	144	3	130000	40000	20	270000
Startle Time (sec)	(y+1)^-1, y*100	Normal	144	3	85000	40000	20	135000
Period 4, Light to Dark								
Startle Magnitude (mm)	(y+1)^-0.14, y*1000	Normal	144	3	100000	40000	20	180000
Startle Time (sec)	(y+1)^-0.58, y*100	Normal	144	3	95000	40000	10	165000
Period 1, Light								
Swimming Bouts (per sec)	(y+1)^4.87, y*10	Normal	144	3	120000	45000	20	225000
Swimming Bout Duration (sec)	(y+1)^-5.3, y*1000	Normal	144	3	100000	45000	20	165000

Swimming Bout Speed (mm/s)	(y+1)^-0.75, y*10000	Normal	144	3	70000	40000	25	90000
Swimming Bout Turning Angle	(y+1)^2.25, y*1000	Normal	144	3	60000	40000	15	60000
Total Distance Traveled (mm)	(y+1)^0.025, y*1000	Normal	144	3	55000	35000	20	60000
Total Time Swimming (sec)	(y+1)^0.65, y*10	Normal	144	3	150000	55000	20	285000
Overall Step Length (mm)	(y+1)^-9.15, y*1000	Normal	144	3	130000	45000	15	255000
Overall Step Length Variation	(y+1)^-4.37, y*1000	Normal	144	3	130000	55000	20	225000
Overall Turning Angle	(y+1)^5.7, y*100	Normal	144	3	90000	40000	20	150000
Overall Turning Angle Variation	(y+1)^-0.34, y*1000	Normal	144	3	100000	40000	20	180000
Period 2, Dark								
Swimming Bouts (per sec)	(y+1)^4.1, y*10	Normal	144	3	150000	40000	20	330000
Swimming Bout Duration (sec)	(y+1)^-4.9, y*1000	Normal	144	3	75000	55000	15	60000
Swimming Bout Speed (mm/s)	(y+1)^0.45, y*1000	Normal	144	3	70000	40000	15	90000
Swimming Bout Turning Angle	(y+1)^12.9, y*10000	Normal	144	3	60000	40000	15	60000
Total Distance Traveled (mm)	(y+1)^0.77	Normal	144	3	65000	35000	20	90000
Total Time Swimming (sec)	(y+1)^2.5, y/10000	Normal	144	3	60000	40000	10	60000
Overall Step Length (mm)	(y+1)^-0.45, y*1000	Normal	144	3	80000	40000	15	120000
Overall Step Length Variation	(y+1)^-2.6, y*1000	Normal	144	3	85000	55000	25	90000
Overall Turning Angle	(y+1)^10.2, y*100	Normal	144	3	60000	40000	15	60000
Overall Turning Angle Variation	(y+1)^-1.17, y*1000	Normal	144	3	70000	40000	20	90000
Period 3, Light								
Swimming Bouts (per sec)	(y+1)^4.35	Normal	144	3	60000	40000	15	60000
Swimming Bout Duration (sec)	(y+1)^-8.4, y*1000	Normal	144	3	120000	45000	20	225000

Swimming Bout Speed (mm/s)	(y+1)^-2.1, y*10000	Normal	144	3	85000	60000	25	75000
Swimming Bout Turning Angle	(y+1)^1.8, y*1000	Normal	144	3	65000	40000	15	75000
Total Distance Traveled (mm)	(y+1)^-0.01, y*10000	Normal	144	3	55000	35000	10	60000
Total Time Swimming (sec)	(y+1)^0.66, y*10	Normal	144	3	125000	55000	20	210000
Overall Step Length (mm)	(y+1)^-11, y*1000	Normal	144	3	150000	40000	20	330000
Overall Step Length Variation	(y+1)^-7.7, y*1000	Normal	144	3	125000	55000	20	210000
Overall Turning Angle	(y+1)^3.6, y*100	Normal	144	3	60000	40000	15	60000
Overall Turning Angle Variation	(y+1)^0.6, y*1000	Normal	144	3	65000	40000	20	75000
Period 4, Dark								
Swimming Bouts (per sec)	(y+1)^4	Normal	144	3	120000	40000	20	240000
Swimming Bout Duration (sec)	(y+1)^-7.93, y*1000	Normal	144	3	95000	40000	20	165000
Swimming Bout Speed (mm/s)	(y+1)^0.7, y*100	Normal	144	3	120000	40000	20	240000
Swimming Bout Turning Angle	(y+1)^1.84, y*1000	Normal	144	3	95000	40000	20	165000
Total Distance Traveled (mm)	(y+1)^0.8	Normal	144	3	75000	40000	20	105000
Total Time Swimming (sec)	(y+1)^2.53, y/1000	Normal	144	3	125000	40000	20	255000
Overall Step Length (mm)	(y+1)^-0.32, y*1000	Normal	144	3	130000	40000	20	270000
Overall Step Length Variation	(y+1)^-1.55, y*1000	Normal	144	3	400000	45000	20	1065000
Overall Turning Angle	(y+1)^1.6, y*1000	Normal	144	3	140000	40000	20	300000
Overall Turning Angle Variation	(y+1)^-1.4, y*1000	Normal	144	3	110000	40000	20	210000

Table S6. Distributions and priors for parameters in models used to determine differences in treatments for locomotion behavior responses.

Model Table	Residual	Residual Variance	Batch Effect	Batch Effect Variance
S6	$\varepsilon \sim Normal(0, \sigma_\varepsilon^2)$	$\frac{1}{\sigma_\varepsilon^2} = \tau_j \sim I - Gamma(0.0001, 0.0001)$	$\varepsilon \sim Normal(0, \sigma_\varepsilon^2)$	$\frac{1}{\sigma_\varepsilon^2} = \tau_j \sim I - Gamma(0.01, 0.01)$
S7	$\varepsilon \sim Normal(0, \sigma_\varepsilon^2)$	$\sqrt{\sigma_\varepsilon^2} = \sigma_\varepsilon \sim U(0,01,1000)$	$\varepsilon \sim Normal(0, \sigma_\varepsilon^2)$	$\sqrt{\sigma_\varepsilon^2} = \sigma_\varepsilon \sim U(0,01,1000)$
S8	$\varepsilon \sim Student's\ T(0, \sigma_\varepsilon^2, df)$	$\frac{1}{\sigma_\varepsilon^2} = \tau_j \sim I - Gamma(0.0001, 0.0001)$	$\varepsilon \sim Student's\ T(0, \sigma_\varepsilon^2, df)$	$\frac{1}{\sigma_\varepsilon^2} = \tau_j \sim I - Gamma(0.01, 0.01)$

Table S7. Normal distribution OpenBUGS model containing treatment and time of assay effects and a random batch effect used to analyze locomotion behavior endpoints.

```
#inits<-function(){
#   list(batch.eff=runif(N2,-1000,1000),tau=runif(1,0,10),tau.a=runif(1,0,10))}
#inits()
model;
{
	for(i in 1:N){
	y[i]~dnorm(mu[i],tau)
	mu[i]<-mean+trt.eff[trt[i]]+time[i]*betta_mfn+batch.eff[batchid[i]]
	}
	mean~dnorm(0,1.0E-6)
#make covariate effect priors
	#time
	betta_mfn~dnorm(0,0.0001)
#make fixed main effect priors
	trt.eff[1]<-0
	for (i in 2:5){
	trt.eff[i]~dnorm(0,1.0E-6)
	}
#make random effect of batch priors
	for (i in 1:N2){
	batch.eff[i]~dnorm(0,tau.a)
	}
#predict estimates
#cell means models
	for(j in 1:5){
	Trt.mean[j]<-mean+trt.eff[j]
	}
#initial values
	var<-1/tau
	var.a<-1/tau.a
	tau~dgamma(0.0001,0.0001)
	tau.a~dgamma(0.01,0.01)
#difference calculations
	trt1<-Trt.mean[1]#sco salmon-fed ctl
	trt2<-Trt.mean[2]#sco tuna/hg fed
	trt3<-Trt.mean[3]#sco salmon-fed pcb40
	trt4<-Trt.mean[4]#nbh salmon-fed ctl
	trt5<-Trt.mean[5]#nbh salmon-fed pcb40
	diftrt2_1<-trt2-trt1
	pvaltrt2_1<-step(diftrt2_1)
	diftrt3_1<-trt3-trt1
	pvaltrt3_1<-step(diftrt3_1)
	diftrt3_2<-trt3-trt2
	pvaltrt3_2<-step(diftrt3_2)
	diftrt4_1<-trt4-trt1
	pvaltrt4_1<-step(diftrt4_1)
```

```
        diftrt5_4<-trt5-trt4
        pvaltrt5_4<-step(diftrt5_4)
        diftrt3_5<-trt3-trt5
        pvaltrt3_5<-step(diftrt3_5)
#ratio calculations
        ratiotrt2_1<-trt2/trt1
        ratiotrt3_1<-trt3/trt1
        ratiotrt3_2<-trt3/trt2
        ratiotrt4_1<-trt4/trt1
        ratiotrt5_4<-trt5/trt4
        ratiotrt3_5<-trt3/trt5
#posterior model checking, generate new obs based on model params mu, tau. assume normal dist
  for( i in 1 : N ) {
    ypred[i] ~ dnorm(mu[i],tau)
  }
#generate individual level predictions
    ypred_1 ~ dnorm(trt1,tau)#approximation of the individual observation, using average for other factors in
the model.
    ypred_2 ~ dnorm(trt2,tau)#randomly selected individual
    ypred_3 ~ dnorm(trt3,tau)
    ypred_4 ~ dnorm(trt4,tau)
    ypred_5 ~ dnorm(trt5,tau)
#compute residuals using the kurtosis formula for both orig data (e) and rep data
  for( i in 1 : N ) {
    e[i]<-y[i]-mu[i]
    }
 SSE<-inprod(e[],e[])#sum of squares which is e squared
 ku<-sum(e[]) #sum up all values, there is one for each data point
 kpred<-sum(ypred[])
difs<-kpred-ku #find difference
difpval<-step(difs) #count how many times the rep data is larger than orig data
}
```

Table S8. Normal distribution OpenBUGS model containing treatment and time of assay effects and a random batch effect using uniform tau prior used to analyze locomotion behavior endpoints.

```
#inits<-function(){
#  list(batch.eff=runif(N2,-1000,1000),sdev=runif(1,0.01,1000),sdev.a=runif(1,0.01,1000))}
#inits()
model;
{
	for(i in 1:N){
	y[i]~dnorm(mu[i],tau)
	mu[i]<-mean+trt.eff[trt[i]]+time[i]*betta_mfn+batch.eff[batchid[i]]
	}
	mean~dnorm(0,1.0E-6)
#make covariate effect priors
	#time
	betta_mfn~dnorm(0,0.0001)
#make fixed main effect priors
	trt.eff[1]<-0
	for (i in 2:5){
	trt.eff[i]~dnorm(0,1.0E-6)
	}
#make random effect of batch priors
	for (i in 1:N2){
	batch.eff[i]~dnorm(0,tau.a)
	}
#predict estimates
#cell means models
	for(j in 1:5){
	Trt.mean[j]<-mean+trt.eff[j]
	}
#initial values
	sdev~dunif(0.01,1000)
	sdev.a~dunif(0.01,1000)
	var<-pow(sdev,2)
	var.a<-pow(sdev.a,2)
	tau<-pow(sdev,-2)
	tau.a<-pow(sdev.a,-2)
#difference calculations
	trt1<-Trt.mean[1]#sco salmon-fed ctl
	trt2<-Trt.mean[2]#sco tuna/hg fed
	trt3<-Trt.mean[3]#sco salmon-fed pcb40
	trt4<-Trt.mean[4]#nbh salmon-fed ctl
	trt5<-Trt.mean[5]#nbh salmon-fed pcb40
	diftrt2_1<-trt2-trt1
	pvaltrt2_1<-step(diftrt2_1)
	diftrt3_1<-trt3-trt1
	pvaltrt3_1<-step(diftrt3_1)
	diftrt3_2<-trt3-trt2
	pvaltrt3_2<-step(diftrt3_2)
	diftrt4_1<-trt4-trt1
```

```
          pvaltrt4_1<-step(diftrt4_1)
          diftrt5_4<-trt5-trt4
          pvaltrt5_4<-step(diftrt5_4)
          diftrt3_5<-trt3-trt5
          pvaltrt3_5<-step(diftrt3_5)
#ratio calculations
          ratiotrt2_1<-trt2/trt1
          ratiotrt3_1<-trt3/trt1
          ratiotrt3_2<-trt3/trt2
          ratiotrt4_1<-trt4/trt1
          ratiotrt5_4<-trt5/trt4
          ratiotrt3_5<-trt3/trt5
#posterior model checking, generate new obs based on model params mu, tau. assume normal dist
   for( i in 1 : N ) {
     ypred[i] ~ dnorm(mu[i],tau)
   }
#generate individual level predictions
     ypred_1 ~ dnorm(trt1,tau)#approximation of the individual observation
     ypred_2 ~ dnorm(trt2,tau)#randomly selected individual
     ypred_3 ~ dnorm(trt3,tau)
     ypred_4 ~ dnorm(trt4,tau)
     ypred_5 ~ dnorm(trt5,tau)

#compute residuals using the kurtosis formula for both orig data (e) and rep data
   for( i in 1 : N ) {
     e[i]<-y[i]-mu[i]
    }
  SSE<-inprod(e[],e[])#sum of squares which is e squared
  ku<-sum(e[]) #sum up all values, there is one for each data point
  kpred<-sum(ypred[])
difs<-kpred-ku #find difference
difpval<-step(difs) #count how many times the rep data is larger than orig data
}
```

Table S9. Student's t distribution OpenBUGS model containing treatment and time of assay main effects and a random batch effect used to analyze locomotion behavior endpoints.

```
#inits<-function(){
#  list(batch.eff=runif(N2,-
1000,1000),df=runif(1,3,30),df.a=runif(1,3,30),tau=runif(1,0,10),tau.a=runif(1,0,10))}
#inits()
model;
{
	for(i in 1:N){
	y[i]~dt(mu[i],tau,df)
	mu[i]<-mean+trt.eff[trt[i]]+time[i]*betta_mfn+batch.eff[batchid[i]]
	}
	mean~dnorm(0,1.0E-6)
#make covariate effect priors
	#time
	betta_mfn~dnorm(0,0.0001)
#make fixed main effect priors
	trt.eff[1]<-0
	for (i in 2:5){
	trt.eff[i]~dnorm(0,1.0E-6)
	}
#make random effect of batch priors
	for (i in 1:N2){
	batch.eff[i]~dt(0,tau.a,df.a)
	}
#predict estimates
#cell means models
	for(j in 1:5){
	Trt.mean[j]<-mean+trt.eff[j]
	}
#initial values
	df~dunif(3,30)
	df.a~dunif(3,30)
	var<-1/tau
	var.a<-1/tau.a
	tau~dgamma(0.0001,0.0001)
	tau.a~dgamma(0.01,0.01)
#difference calculations
	trt1<-Trt.mean[1]#sco salmon-fed ctl
	trt2<-Trt.mean[2]#sco tuna/hg fed
	trt3<-Trt.mean[3]#sco salmon-fed pcb40
	trt4<-Trt.mean[4]#nbh salmon-fed ctl
	trt5<-Trt.mean[5]#nbh salmon-fed pcb40
	diftrt2_1<-trt2-trt1
	pvaltrt2_1<-step(diftrt2_1)
	diftrt3_1<-trt3-trt1
	pvaltrt3_1<-step(diftrt3_1)
	diftrt3_2<-trt3-trt2
```

```
        pvaltrt3_2<-step(diftrt3_2)
        diftrt4_1<-trt4-trt1
        pvaltrt4_1<-step(diftrt4_1)
        diftrt5_4<-trt5-trt4
        pvaltrt5_4<-step(diftrt5_4)
        diftrt3_5<-trt3-trt5
        pvaltrt3_5<-step(diftrt3_5)
#ratio calculations
        ratiotrt2_1<-trt2/trt1
        ratiotrt3_1<-trt3/trt1
        ratiotrt3_2<-trt3/trt2
        ratiotrt4_1<-trt4/trt1
        ratiotrt5_4<-trt5/trt4
        ratiotrt3_5<-trt3/trt5
#posterior model checking, generate new obs based on model params mu, tau. assume normal dist
  for( i in 1 : N ) {
    ypred[i] ~ dt(mu[i],tau,df)
  }
#generate individual level predictions
    ypred_1 ~ dt(trt1,tau,df)#approximation of the individual observation, using average for other factors in the model.
    ypred_2 ~ dt(trt2,tau,df)#randomly selected individual
    ypred_3 ~ dt(trt3,tau,df)
    ypred_4 ~ dt(trt4,tau,df)
    ypred_5 ~ dt(trt5,tau,df)
#compute residuals using the kurtosis formula for both orig data (e) and rep data
  for( i in 1 : N ) {
    e[i]<-y[i]-mu[i]
   }
 SSE<-inprod(e[],e[])#sum of squares which is e squared
 ku<-sum(e[]) #sum up all values, there is one for each data point
 kpred<-sum(ypred[])
difs<-kpred-ku #find difference
difpval<-step(difs) #count how many times the rep data is larger than orig data
}
```

Table S10. Normal distribution OpenBUGS model containing treatment, time of assay and days post hatch (dpf) effects used to analyze feeding behavior endpoints.

```
#inits<-function(){
#  list(tau=runif(1,0,10))
#}
model;
{
	for(i in 1:N){
	y[i]~dnorm(mu[i],tau)
	mu[i]<-mean+trt.eff[trt[i]]+time[i]*betta_mfn+dpf[i]*betta_dpf
	}
	mean~dnorm(0,1.0E-6)
#make covariate effect priors
	#time
	betta_mfn~dnorm(0,0.0001)
	#dpf
	#independent gaussian priors for the linear covariate
	betta_dpf~dnorm(0,0.0001)
#make fixed main effect priors
	trt.eff[1]<-0
	for (i in 2:5){
	trt.eff[i]~dnorm(0,1.0E-6)
	}
#back transform the outputs
#cell means models
	for(j in 1:5){
	Trt.mean[j]<-mean+trt.eff[j]
	}
#initial values
	tau~dgamma(0.0001,0.0001)
	var<-1/tau
	trt1<-Trt.mean[1]#sco salmon-fed ctl
	trt2<-Trt.mean[2]#sco tuna/hg fed
	trt3<-Trt.mean[3]#sco salmon-fed pcb40
	trt4<-Trt.mean[4]#nbh salmon-fed ctl
	trt5<-Trt.mean[5]#nbh salmon-fed pcb40
	diftrt2_1<-trt2-trt1
	pvaltrt2_1<-step(diftrt2_1)
	diftrt3_1<-trt3-trt1
	pvaltrt3_1<-step(diftrt3_1)
	diftrt3_2<-trt3-trt2
	pvaltrt3_2<-step(diftrt3_2)
	diftrt4_1<-trt4-trt1
	pvaltrt4_1<-step(diftrt4_1)
	diftrt5_4<-trt5-trt4
	pvaltrt5_4<-step(diftrt5_4)
	#diftrt6_4<-trt6-trt4
	#pvaltrt6_4<-step(diftrt6_4)
```

```
        #diftrt6_5<-trt6-trt5
        #pvaltrt6_5<-step(diftrt6_5)
        diftrt3_5<-trt3-trt5
        pvaltrt3_5<-step(diftrt3_5)
#ratio calculations
        ratiotrt2_1<-trt2/trt1
        ratiotrt3_1<-trt3/trt1
        ratiotrt3_2<-trt3/trt2
        ratiotrt4_1<-trt4/trt1
        ratiotrt5_4<-trt5/trt4
        #ratiotrt6_4<-trt6/trt4
        #ratiotrt6_5<-trt6/trt5
        ratiotrt3_5<-trt3/trt5
#posterior model checking, generate new obs based on model params mu, tau. assume normal dist
  for( i in 1 : N ) {
    ypred[i] ~ dnorm(mu[i],tau)
  }
#generate individual level predictions
    ypred_1 ~ dnorm(trt1,tau)#approximation of the individual observation, using average for other factors in
the model.
    ypred_2 ~ dnorm(trt2,tau)#randomly selected individual
    ypred_3 ~ dnorm(trt3,tau)
    ypred_4 ~ dnorm(trt4,tau)
    ypred_5 ~ dnorm(trt5,tau)
    #ypred_6 ~ dnorm(trt6,tau)
#compute residuals using the kurtosis formula for both orig data (e) and rep data
  for( i in 1 : N ) {
    e[i]<-y[i]-mu[i]
   }
 SSE<-inprod(e[],e[])#sum of squares which is e squared
 ku<-sum(e[]) #sum up all values, there is one for each data point
 kpred<-sum(ypred[])
difs<-kpred-ku #find difference
difpval<-step(difs) #count how many times the rep data is larger than orig data
}
```

Table S11. Binomial distribution OpenBUGS model containing treatment, time of assay and days post hatch (dpf) effects used to analyze feeding endpoints.

```
#inits<-function(){
#  list(betta_mfn=runif(1,0,5),Trt.mean=runif(5,0,5),tau=runif#(1,0,10),betta_dpf=runif(1,0,5))
#}
#inits()
model
{
	for( i in 1 : N ) {
	y[i] ~ dbin(p[i],bs[i])
	e[i]~dnorm(0,tau)
	logit(p[i]) <-time[i]*betta_mfn+dpf[i]*betta_dpf+Trt.mean[trt[i]]+e[i]
	}
#set priors
	tau ~ dgamma(0.01,0.01)
	var<-1/tau
#make covariate effect priors
	#time
	betta_mfn~dnorm(0,0.0001)
	#dpf
	#independent gaussian priors for the linear covariate
	betta_dpf~dnorm(0,0.0001)
#make fixed main effect priors
	for (i in 1:5){
	Trt.mean[i]~dnorm(0,1.0E-6)
	}
#back transform the outputs
#cell means models
	for(j in 1:5){
	trt.eff[j]<-Trt.mean[j]-Trt.mean[1]
	}
#other values
	trt1<-1/(1+exp(-Trt.mean[1]))#sco salmon-fed ctl, back transformed trt mean, in the scale of the
binomial prob. the probability of being attacked by the average population. do not back transformed
	trt2<-1/(1+exp(-Trt.mean[2]))#sco tuna/hg fed
	trt3<-1/(1+exp(-Trt.mean[3]))#sco salmon-fed pcb40
	trt4<-1/(1+exp(-Trt.mean[4]))#nbh salmon-fed ctl
	trt5<-1/(1+exp(-Trt.mean[5]))#nbh salmon-fed pcb40

	diftrt2_1<-Trt.mean[2]-Trt.mean[1]#compare on linear scale logit
	pvaltrt2_1<-step(diftrt2_1)
	diftrt3_1<-Trt.mean[3]-Trt.mean[1]
	pvaltrt3_1<-step(diftrt3_1)
	diftrt3_2<-Trt.mean[3]-Trt.mean[2]
	pvaltrt3_2<-step(diftrt3_2)
	diftrt4_1<-Trt.mean[4]-Trt.mean[1]
	pvaltrt4_1<-step(diftrt4_1)
	diftrt5_4<-Trt.mean[5]-Trt.mean[4]
	pvaltrt5_4<-step(diftrt5_4)
```

```
	diftrt3_5<-Trt.mean[3]-Trt.mean[5]
	pvaltrt3_5<-step(diftrt3_5)
#ratio calculations
	ratiotrt2_1<-trt2/trt1 #use the back transformed scale
	ratiotrt3_1<-trt3/trt1
	ratiotrt3_2<-trt3/trt2
	ratiotrt4_1<-trt4/trt1
	ratiotrt5_4<-trt5/trt4
	ratiotrt3_5<-trt3/trt5
#posterior model checking, generate new obs based on model params
	for( i in 1 : N ) {
		ypred[i] ~ dbin(p[i],bs[i])
	}
#generate individual level predictions
	#need to estimate error for each group
	for(j in 1:5){
	ee[j]~dnorm(0,tau)
	}
	ypred_1 <- 1/(1+exp(-(Trt.mean[1]+ee[1]))) #probability of bs capture by a random individual in trt1
	ypred_2 <- 1/(1+exp(-(Trt.mean[2]+ee[2])))
	ypred_3 <- 1/(1+exp(-(Trt.mean[3]+ee[3])))
	ypred_4 <- 1/(1+exp(-(Trt.mean[4]+ee[4])))
	ypred_5 <- 1/(1+exp(-(Trt.mean[5]+ee[5])))

}
```

Table S12. A list of all parameters included in the individual-based model, units, equation reference and references (mm = millimeter, m = meter, d = day, g = gram, ◦C = Celsius, J = joule, # = count, s = sec, hr = hour, µg = microgram, O_2 = oxygen, W = weight, L = length, ml = milliliter).

Variable	Value	Units	Explanation	Reference
			**Initialize larva**	
Number of fish	2500	#	Number of larva	Smith et al. 2002
Mean Length	5.96	mm	Mean length	Marteinsdottir and Able 1992
Mean Stdev of Length	0.4	mm	Standard deviation of length	Marteinsdottir and Able 1992
Length max	8	mm	Maximum length	Estimated
Length min	5	mm	Minimum length	Estimated
Length at which fish exists model	24	mm	Size at exit	Abraham 1985
			**Time & physical**	
Initial day of model	100	day	Julian date	
Number of days model ran	100	day		
Volume	1000	m^3	Volume of the aquatic habitat modeled	
			**Yolk-sac growth**	
Yolk-sac growth	0.40	mm/d	Growth of yolk-sac larvae	Marteinsdottir and Able 1992
Length exogenous feeding begins	4	days	Days until start of feeding	Estimated
W_L a parameter	0.0000015	g dry	Length-weight function intercept	Kneib and Parker 1991
W-L b parameter	3.25	NA	Length-weight function slope	Kneib and Parker 1991
			**Bioenergetics (from Deslauriers et al. 2017 unless otherwise noted)**	
Ca	0.2	g/g/d	Intercept of the mass dependence function for consumption	
Cb	-0.25	NA	Slope of the mass dependence function for consumption	
CQ	2.22	◦C	Temperature-dependent coefficient of consumption (approximates Q10)	
CTO	27	◦C	Optimal temperature for consumption	
CTM	34	◦C	Maximum consumption temperature	
Ra	0.02	gO_2/g/d	Intercept of the mass dependence function for respiration	
Rb	-0.17	NA	Slope of the mass dependence function for respiration	
RQ	2	◦C	Temperature-dependent coefficient of respiration (approximates Q10)	
RTO	29	◦C	Optimal temperature for respiration	

RTM	36	◦C	Maximum respiration temperature	
Act	1.25	NA	Activity multiplier on respiration	
SDA	0.1	NA	Specific dynamic action coefficient	
FA	0.1	NA	Egestion coefficient	
UA	0.06	NA	Excretion coefficient	
ED	3000	J/g wet	Energy density of larvae	
Percent dry	0.2	%	Dry to wet conversion	Estimated
Starvation	75	%	Probability of starvation	Letcher et al. 1996
			**Prey**	
Small prey density	0.0175	#/ml	Copepods	Fleeger et al. 2008
Large prey density	0.008	#/ml	Amphipods	Estimated but based on ostracods in Fleeger et al. 2008
Small prey length	0.485	ml	Copepods	Fulford et al. 2006
Large prey length	0.6	ml	Amphipods	Fulford et al. 2006
Small prey mass	1.215	µg dry	Copepods	Fulford et al. 2006
Large prey mass	3.8	µg dry	Amphipods	Fulford et al. 2006
Large prey energy density	2301.2	J/g wet	Copepods	Hartman and Brandt 1995
Small prey energy density	4125.424	J/g wet	Amphipods	Hartman and Brandt 1995
			**Foraging**	
SSa	0.776	mm/s	Intercept of the fish length to swimming speed function	Letcher et al. 1996
SSb	1.07	NA	Slope of the fish length to swimming speed function	Letcher et al. 1996
Handling Time a	0.264	s	Intercept of the handling time function relative to prey to predator length ratio	Walton et al. 1992
Handling Time b	7.0151	NA	Slope of the handling time function relative to prey to predator length ratio	Walton et al. 1992
Light	12	hr	Active time during the day	Letcher et al. 1996
			**Killifish Predators (Adults)**	
Number of predators	200	#	Number of predators	Calibrated
Mean predator length	45	mm	Mean predator length	Assigned (Age 1 size)
Stdev predator length	2.5	mm	Standard deviation of length	Estimated
Min predator length	25.5	mm	Minimum length	Estimated
Max predator length	96	mm	Maximum length	Valiela et al. 1977
Predator CTM	34	◦C	Maximum temperature for consumption	Madon et al. 2001
Predator CTO	27	◦C	Optimum temperature for consumption	Madon et al. 2001

Predator CQ	2.22	◦C	Temperature-dependent coefficient of consumption (approximates Q10)	Madon et al. 2001
Predator swimming speed	3	Body Lengths	Multiplier on body lengths for distance swum in a second	Cowan et al. 1996
Predator reactive distance	0.8	mm	Reactive distance multiplier	Cowan et al. 1996
			**Calibration**	
Growth	0.31	mm/d	Change in fish length from 0-16 days post hatch	Nacci unpublished data

Table S13. A list of all behavior parameter distributions and resulting multipliers used to assess treatment impacts in the individual based model. Posterior distributions are from the individual level predicted responses and multipliers were generated from back transformed values. N indicates this behavior was significantly lower than the control, P indicates this behavior was significantly higher than the control.

Killifish			Individual Level Distribution					Multipliers			
Group	Chemical	Variable	Mean	SD	Minimum	Maximum		Mean	SD	Minimum	Maximum
SCO	Control	Prey Handling Time	27.89	8.186	11.88	43.67		1.000	0.287	0.547	1.986
SCO	Control	Prey Miss Proportion	-2.674	1.308	-5.255	-0.1185		1.000	0.110	0.589	1.105
SCO	Control	Reaction Distance (mm)	167.3	19.05	129.4	204.9		1.000	0.232	0.464	1.510
SCO	Control	Swimming Bout Speed (mm/s)	137.1	27.9	82.41	191.7		1.000	0.221	0.644	1.698
SCO	Control	Total Time Swimming (sec)	58.94	16.72	26.14	91.75		0.547	0.168	0.194	0.943
SCO	MeHg	Prey Handling Time	28.51	8.2	12.4	44.64		0.972	0.281	0.530	1.924
SCO	MeHg	Prey Miss Proportion	-3.305	1.328	-5.952	-0.7121		1.047	0.070	0.746	1.108
SCO	MeHg	Reaction Distance (mm)	177.7	19.1	139.9	215.3	p	1.151	0.226	0.619	1.643
SCO	MeHg	Swimming Bout Speed (mm/s)	144.4	27.91	89.75	199.1		0.936	0.194	0.614	1.547
SCO	MeHg	Total Time Swimming (sec)	53.25	16.69	20.48	85.98		0.481	0.163	0.142	0.869
SCO	PCB126	Prey Handling Time	17.94	8.278	1.574	34.12	p	1.662	0.827	0.756	7.003
SCO	PCB126	Prey Miss Proportion	-1.346	1.327	-3.963	1.251	p	0.826	0.211	0.247	1.090
SCO	PCB126	Reaction Distance (mm)	172.3	19.32	134.6	209.9		1.078	0.230	0.540	1.575
SCO	PCB126	Swimming Bout Speed (mm/s)	142.8	28.13	87.5	197.8		0.951	0.203	0.619	1.591
SCO	PCB126	Total Time Swimming (sec)	48	16.85	14.91	81.09	n	0.425	0.161	0.094	0.808
NBH	Control	Prey Handling Time	27.89	8.162	11.74	43.84		1.000	0.287	0.545	2.004
NBH	Control	Prey Miss Proportion	-3.003	1.308	-5.589	-0.4375		1.000	0.083	0.657	1.076
NBH	Control	Reaction Distance (mm)	179.9	18.97	142.5	216.9		1.000	0.190	0.557	1.414
NBH	Control	Swimming Bout Speed (mm/s)	141.5	27.87	86.93	196		1.000	0.212	0.654	1.670
NBH	Control	Total Time Swimming (sec)	51.82	16.7	19.06	84.56		0.466	0.163	0.130	0.851
NBH	PCB126	Prey Handling Time	23.22	8.243	6.883	39.16	p	1.238	0.424	0.637	2.917
NBH	PCB126	Prey Miss Proportion	-2.261	1.323	-4.893	0.314		0.933	0.135	0.457	1.072

NBH	PCB126	Reaction Distance (mm)	172	19.27	133.6	210	0.907	0.198	0.446	1.339
NBH	PCB126	Swimming Bout Speed (mm/s)	134.3	28.12	79.02	189.3	1.073	0.243	0.682	1.856
NBH	PCB126	Total Time Swimming (sec)	49.88	16.81	16.94	82.89	0.443	0.161	0.111	0.830

Table S14. Posterior distributions for all model parameters and each behavioral endpoint.

Submitted this table as a text tab separated file.

Table S15. Significant results of the treatment effects on Atlantic killifish larvae behavior after exposure to sublethal levels of MeHg and PCB126. Presented for each behavior endpoint and treatment is the mean (original or back-transformed), transformed mean, P-value in parentheses, and pattern of significant trends. Trends are based on original mean trends. P-values and trends are reported in the following order: SCO-Ctrl vs SCO-Hg, SCO-Ctrl vs SCO-PCB, SCO-Ctrl vs NBH-Ctrl, SCO-PCB vs NBH-PCB, NBH-Ctrl vs NBH-PCB (Neg = significant negative trend, Pos = significant positive trend, □ = no significant trend, HMM = Hidden Markov Chain model endpoint).

Parameter	SCO-Ctrl	SCO-Hg	SCO-PCB	NBH-Ctrl	NBH-PCB	Significant Trends
VMR Assay						
Period 2, Light to Dark						
Startle Magnitude (mm)	53.1345	59.8116	52.3395	68.6335	63.3233	
	487.5	477.4	488.8	465.9	472.6	
	(0.3196)	(0.9036)	(0.0360)	(0.1214)	(0.5232)	□ □ Pos □ □
Period 4, Light to Dark	52.2579	53.0612	50.9493	57.2169	66.7569	
Startle Magnitude (mm)	573.2	572	575.2	566.1	554.2	
	(0.8874)	(0.8288)	(0.4220)	(0.0232)	(0.1919)	□ □ □ Neg □
Period 1, Light						
Swimming Bouts (per sec)	1.4819	1.5468	1.2431	1.5020	1.4358	
	836.7	948.8	511.2	870.3	763.7	
	(0.1182)	(0.0)	(0.6360)	(0.0009)	(0.1418)	□ Neg □ Neg □
Swimming Bout Duration (sec)	0.3002	0.2629	0.2542	0.2749	0.2619	
	248.7	290.2	301.1	276.1	291.5	
	(0.1124)	(0.0464)	(0.2898)	(0.7192)	(0.5566)	□ Neg □ □ □
Swimming Bout Speed (mm/s)	6.5159	6.3247	5.7085	6.2343	6.3508	
	2203	2246	2399	2267	2240	
	(0.5312)	(0.0058)	(0.3576)	(0.0268)	(0.7044)	□ Neg □ Neg □
Total Distance Traveled (mm)	1793.29	1570.01	1123.74	1678.03	1468.77	
	1206	1202	1192	1204	1200	

	(0.2974)	(0.0002)	(0.5050)	(0.0264)	(0.3612)	□ Neg □ Neg □
Total Time Swimming (sec)	274.1170	248.8296	193.3380	260.8265	234.9453	
	385.2	361.8	307.3	373	348.6	
	(0.2366)	(0.0002)	(0.5358)	(0.0443)	(0.2256)	□ Neg □ Neg □
Overall Step Length (mm)	0.0994	0.0890	0.0649	0.0935	0.0835	
	420.3	458.4	562.6	441.5	480.1	
	(0.3298)	(0.0006)	(0.5848)	(0.0410)	(0.3312)	□ Neg □ Neg □
Overall Step Length Variation	0.2049	0.1952	0.1677	0.2062	0.1937	
	442.8	458.8	507.8	440.8	461.3	
	(0.4754)	(0.0048)	(0.9284)	(0.0464)	(0.3688)	□ Neg □ Neg □
Overall Turning Angle Variation	1.3142	1.2338	1.5346	1.2971	1.2882	
	751.8	760.9	728.9	753.7	754.7	
	(0.3578)	(0.0220)	(0.8462)	(0.0112)	(0.9192)	□ Pos □ Pos □
Period 2, Dark						
Swimming Bouts (per sec)	2.2416	2.3658	1.9063	2.2950	2.2384	
	1242	1449	793.8	1328	1237	
	(0.0688)	(0.0001)	(0.4440)	(0.0002)	(0.430)	□ Neg □ Neg □
Swimming Bout Duration (sec)	0.3432	0.3025	0.3778	0.3158	0.3061	
	235.6	273.9	208	260.6	270.2	
	(0.0712)	(0.1895)	(0.2334)	(0.0045)	(0.6512)	□ □ □ Pos □
Overall Turning Angle	-0.0051	-0.0028	0.0023	-0.0009	0.0022	
	94.96	97.15	102.4	99.1	102.3	
	(0.5550)	(0.0470)	(0.2654)	(0.9728)	(0.3944)	□ Pos □ □ □
Period 3, Light						
Swimming Bouts (per sec)	2.9236	3.0012	2.4600	2.9255	2.8737	
	382.4	416.4	221.3	383.2	361.7	
	(0.3224)	(0.0)	(0.9792)	(0.0002)	(0.5366)	□ Neg □ Neg □
Swimming Bout Duration (sec)	0.1563	0.1256	0.1268	0.1321	0.1375	
	295.3	370.1	366.8	352.8	338.8	
	(0.0168)	(0.0228)	(0.0634)	(0.3758)	(0.6542)	Neg Neg □ □ □

Total Distance Traveled (mm)	1738.8938<br>9281<br>(0.0588)	1357.4067<br>9304<br>(0.0004)	1060.2124<br>9327<br>(0.1918)	1463.5928<br>9297<br>(0.0092)	1511.6317<br>9294<br>(0.8392)	□ Neg □ Neg □
Total Time Swimming (sec)	275.6974<br>409<br>(0.0496)	231.1920<br>364.3<br>(0.0002)	191.4978<br>321.9<br>(0.1236)	240.7212<br>374.1<br>(0.0126)	247.0143<br>380.5<br>(0.780)	Neg Neg □ Neg □
Overall Step Length (mm)	0.0948<br>369.4<br>(0.0508)	0.0752<br>450.3<br>(0.0006)	0.0620<br>516.1<br>(0.1834)	0.0811<br>424.1<br>(0.0130)	0.0844<br>410.3<br>(0.7398)	□ Neg □ Neg □
Overall Step Length Variation	0.1940<br>255.3<br>(0.0876)	0.1722<br>294.2<br>(0.0024)	0.1565<br>326.4<br>(0.3310)	0.1813<br>277.3<br>(0.0328)	0.1818<br>276.4<br>(0.9690)	□ Neg □ Neg □
Overall Turning Angle Variation	1.3954<br>1689<br>(0.5788)	1.4524<br>1713<br>(0.0156)	1.6561<br>1797<br>(0.8142)	1.4191<br>1699<br>(0.0022)	1.3179<br>1656<br>(0.3350)	□ Pos □ Pos □
Period 4, Dark						
Swimming Bouts (per sec)	3.6596<br>471.4<br>(0.1446)	3.8141<br>537.1<br>(0.0)	3.1062<br>284.3<br>(0.6766)	3.7049<br>490<br>(0.0001)	3.6593<br>471.3<br>(0.6808)	□ Neg □ Neg □
Swimming Bout Duration (sec)	0.2132<br>216<br>(0.0596)	0.1882<br>254.7<br>(0.1262)	0.2376<br>184.4<br>(0.1592)	0.1943<br>244.6<br>(0.0035)	0.1930<br>246.7<br>(0.920)	□ □ □ Pos □
Total Distance Traveled (mm)	5464.0032<br>977.4<br>(0.0240)	4659.5653<br>860.5<br>(0.4388)	5184.4966<br>937.2<br>(0.3494)	5132.6770<br>929.7<br>(0.8470)	5115.4270<br>927.2<br>(0.9570)	Neg □ □ □ □
Overall Step Length (mm)	0.3034<br>918.7<br>(0.020)	0.2584<br>929.1<br>(0.3978)	0.2867<br>922.5<br>(0.3298)	0.2845<br>923<br>(0.8648)	0.2837<br>923.2<br>(0.9572)	Neg □ □ □ □

Overall Step Length Variation	0.3931	0.3562	0.3638	0.3932	0.3938	
	598.2	623.6	618.2	598.1	597.7	
	(0.0444)	(0.1142)	(0.9948)	(0.1124)	(0.9770)	Neg □ □ □ □
Locomotion Assay						
Swimming Bouts (per sec)	0.6614	0.6489	0.5510	0.6538	0.6217	
	706	685.8	541.8	693.6	643.3	
	(0.5820)	(0.0004)	(0.7370)	(0.0460)	(0.2444)	□ Neg □ Neg □
Swimming Bout Duration (sec)	0.7389	0.6894	0.6973	0.6522	0.6834	
	16.11	17.72	17.45	19.07	17.93	
	(0.2120)	(0.4114)	(0.0218)	(0.7884)	(0.4554)	□ □ Neg □ □
Swimming Bout Turning Angle	-0.0006	0.0019	-0.0130	0.0107	-0.0402	
	398.9	404.7	346.3	396.1	403	
	(0.8572)	(0.4998)	(0.4304)	(0.1922)	(0.0038)	□ □ □ □ Neg
Total Distance Traveled (mm)	998.40	1005.00	966.80	1028.00	899.10	
	465.2	444.8	433	444.3	441.5	
	(0.0739)	(0.0261)	(0.0699)	(0.5896)	(0.8328)	□ Neg □ □ □
Total Time Swimming (sec)	162.3100	142.7790	125.3102	138.0342	131.5586	
	58.94	53.23	47.99	51.82	49.88	
	(0.0588)	(0.0045)	(0.0197)	(0.6506)	(0.5844)	□ Neg Neg □ □
HMM Endpoints						
Medium State						
Step Length (mm)	0.2631	0.2666	0.2673	0.2693	0.2619	
	90.54	83.41	81.92	78.15	93.11	
	(0.2846)	(0.1266)	(0.0701)	(0.0460)	(0.0282)	□ □ □ Pos Neg
Step Length Variation	0.0483	0.0514	0.0516	0.0536	0.0475	
	24.28	22.26	22.12	20.89	24.87	
	(0.2842)	(0.1797)	(0.0794)	(0.0863)	(0.0386)	□ □ □ □ Neg
Turning Angle	0.2329	-0.3253	-0.0783	-0.5140	0.2078	
	2.329	-3.253	-0.7827	-5.14	2.078	
	(0.1170)	(0.3038)	(0.0417)	(0.3454)	(0.0468)	□ □ Neg □ Pos
Turning Angle Variation	1.9057	1.0184	1.3174	1.2779	1.5122	
	49.99	63.35	57.91	58.56	54.95	

	(0.0084)	(0.0646)	(0.0992)	(0.490)	(0.4818)	Neg □ □ □ □
State Transition Probabilities						
Slow -> Slow	0.9094	0.9305	0.8851	0.9570	0.9122	
	126.5	130.4	122.5	136.2	127	
	(0.2398)	(0.1464)	(0.0056)	(0.0985)	(0.0079)	□ □ Pos □ Neg
Medium -> Slow	0.5339	0.6187	0.5556	0.7497	0.5763	
	53.39	61.87	55.56	74.97	57.63	
	(0.1552)	(0.6680)	(0.0006)	(0.6762)	(0.0048)	□ □ Pos □ Neg
Slow -> Medium	0.0575	0.0481	0.0709	0.0277	0.0564	
	24.21	22.11	26.95	16.71	23.97	
	(0.4856)	(0.2776)	(0.0144)	(0.2382)	(0.0174)	□ □ Neg □ Pos
Medium -> Medium	0.3892	0.5603	0.4469	0.6711	0.4445	
	38.92	56.03	44.69	67.11	44.45	
	(0.0258)	(0.3768)	(0.0004)	(0.9704)	(0.0038)	Pos □ Pos □ Neg
Fast -> Slow	0.0051	0.0036	0.0071	0.0023	0.0056	
	69.58	77.34	60.35	85.07	67.38	
	(0.3214)	(0.1630)	(0.0568)	(0.2860)	(0.0290)	□ □ □ □ Pos
Slow -> Fast	0.0226	0.0188	0.0296	0.0124	0.0223	
	15.1	13.75	17.29	11.14	14.98	
	(0.3210)	(0.0592)	(0.0061)	(0.0432)	(0.0072)	□ □ Neg Pos Pos
Medium -> Fast	0.1753	0.1164	0.1567	0.1142	0.1581	
	43.88	57.04	47.59	57.61	47.3	
	(0.0244)	(0.4452)	(0.0224)	(0.9528)	(0.0831)	Neg □ Neg □ □
Fast -> Fast	0.7732	0.7981	0.7748	0.7966	0.7738	
	137.8	155.4	138.9	154.3	138.2	
	(0.0380)	(0.8732)	(0.0654)	(0.9198)	(0.0718)	Pos □ □ □ □
Feeding Assay						
Prey Capture Probability	0.9962	0.9999	0.9986	0.9999	0.9992	
	5.559	9.482	6.576	9.644	7.176	
	(0.0058)	(0.5228)	(0.0032)	(0.7296)	(0.1432)	Pos □ Pos □ □
Prey Handling Time	0.8375	0.8171	1.2669	0.8368	1.0039	
	27.87	28.53	17.93	27.89	23.23	

						□ Pos □ Pos
	(0.6996)	(0.0)	(0.9940)	(0.0227)	(0.0155)	Pos
Capture Attempt Ratio	0.9400	1.0390	1.1956	1.0484	1.1340	
	48.24	45.67	42.1	45.44	43.44	
						Pos Pos Pos □
	(0.0430)	(0.0001)	(0.0226)	(0.4230)	(0.1535)	□
Prey Miss Proportion	0.0649	0.0355	0.2064	0.0474	0.0935	
	-2.667	-3.302	-1.347	-3.001	-2.272	
	(0.0865)	(0.0012)	(0.3366)	(0.0334)	(0.0606)	□ Pos □ Pos □
Reaction Distance (mm)	0.4093	0.4671	0.4372	0.4792	0.4350	
	167.3	177.7	172.3	179.9	171.9	
	(0.0096)	(0.2978)	(0.0012)	(0.9364)	(0.0732)	Pos □ Pos □ □

Table S16. Significantly differentially expressed genes (alpha = 0.05) found in the brains of Atlantic killifish *Fundulus heteroclitus* in this study. Significant trends and FDR value are reported (Ctrl = Control treatment, Neg = significant negative trend, Pos = significant positive trend, SCO = Scorton Creek larvae, NBH = New Bedford Harbor larvae, Hg = methylmercury). Blanks indicate comparison was tested but did not result in a significant difference.

Submitted this table as a text tab separated file.

Table S17. Significantly altered gene pathways (alpha = 0.05) found in the brains of Atlantic killifish *Fundulus heteroclitus* in this study. Significant trends and q-value are reported (Ctrl = Control treatment, Neg = significant negative trend, Pos = significant positive trend, SCO = Scorton Creek larvae, NBH = New Bedford Harbor larvae, Hg = methylmercury). Blanks indicate comparison was tested but did not result in a significant difference.

Submitted this table as a text tab separated file.

Table S18. Significant MeHg treatment patterns shared by differentially expressed genes and behavior endpoints in Atlantic killifish *Fundulus heteroclitus* found in this study. Both the original and opposite behavior endpoint trends are listed (Neg = significant negative trend, Pos = significant positive trend, - = no significant trend, HMM = Hidden Markov Chain model endpoint).

Significant Treatment Pattern	Gene Expression	Behavior Endpoint
Pos	LOC105915521, LOC105916522, LOC105917295, LOC105918273, LOC105922825, LOC105924291, LOC105934237, LOC105936060, LOC110366363, LOC110366373, LOC118559084, LOC118560703, LOC118560704, LOC118562969, LOC118563898, si:ch211-186j3.6	HMM Fast -> Fast Transition Probabilities, Lung Ratio, HMM Medium -> Medium Transition Probabilities, Prey Capture Probability, Reaction Distance (mm)
Neg	klhl6, LOC105915433, LOC105933875, LOC118566104, scamp1, si:dkey-21c1.4	HMM Medium State Turning Angle Variation, HMM Medium -> Fast Transition Probabilities, Overall Step Length Period 4 (mm), Overall Step Length Variation Period 4, Swimming Bout Duration Period 3 (sec), Total Distance Traveled Period 4 (mm), Total Time Swimming Period 3 (sec)

Table S19. Significant PCB126 (PCB) treatment patterns shared by differentially expressed genes and behavior endpoints in Atlantic killifish *Fundulus heteroclitus* found in this study. Both the original and opposite behavior endpoint trends are listed (Ctrl = Control treatment, Neg = significant negative trend, Pos =

significant positive trend, NS = no significant trend, HMM = Hidden Markov Chain model endpoint, TP = Transition Probability).

Submitted this table as a text tab separated file.

Table S20. Significant PCB126 (PCB) treatment patterns shared by gene pathways and behavior endpoints in Scorton Creek (SCO) Atlantic killifish found in this study. Both the original and opposite behavior endpoint trends are listed (Ctrl = Control treatment, Neg = significant negative trend, Pos = significant positive trend, NS = no significant trend, HMM = Hidden Markov Chain model endpoint, TP = Transition Probability).

Submitted this table as a text tab separated file.

Table S21. Summary of PCB126 (PCB) significant treatment patterns found in Atlantic killifish behavior endpoints (Ctrl = control treatment, Tan = significant negative trend compared to control, Blue = significant positive trend compared to control, Black = no significant trend compared to control, HMM = Hidden Markov Chain model endpoint, TP = Transition Probability).

	Significant Treatment Pattern				
Reference Number	SCO-Ctrl vs SCO-PCB	SCO-Ctrl vs NBH-Ctrl	SCO-PCB vs NBH-PCB	NBH-Ctrl vs NBH-PCB	Behavior Endpoint
1	Blue	Black	Blue	Blue	Prey Handling Time
2	Blue	Black	Blue	Black	Prey Miss Proportion, Overall Turning Angle Variation Period 1, Overall Turning Angle Variation Period 3
3	Tan	Black	Tan	Black	Swimming Bouts (per sec), Swimming Bouts Period 1 (per sec), Swimming Bouts Period 2 (per sec), Swimming Bouts Period 3 (per sec), Swimming Bouts Period 4 (per sec), Swimming Bout Speed Period 1 (mm/s), Total Distance Traveled Period 1 (mm), Total Time Swimming Period 1 (sec), Overall Step Length Period 1 (mm), Overall Step Length Variation Period 1, Total Distance Traveled Period 3 (mm), Overall Step Length Period 3 (mm), Overall Step Length Variation Period 3
4	Blue	Black	Black	Black	Overall Turning Angle Period 2
5	Tan	Black	Black	Black	Total Distance Traveled (mm), Swimming Bout Duration Period 1 (sec), Swimming Bout Duration Period 3 (sec)
6	Blue	Blue	Black	Black	Capture Attempt Ratio
7	Tan	Tan	Black	Black	Total Time Swimming (sec)
8	Black	Blue	Black	Black	Prey Capture Probability, Reaction Distance (mm), Startle Magnitude Period 2
9	Black	Tan	Black	Black	Swimming Bout Duration (sec), HMM Medium -> Fast TP
10	Black	Blue	Black	Tan	HMM Slow -> Slow TP, HMM Medium -> Slow TP, HMM Medium -> Medium TP
11	Black	Tan	Black	Blue	HMM Medium State Turning Angle, HMM Slow -> Medium TP

12	Black	Black	Blue	Tan	HMM Medium State Step Length (mm)
13	Black	Black	Black	Blue	HMM Fast -> Slow TP
14	Black	Black	Black	Tan	HMM Medium State Step Length Variation, Swimming Bout Turning Angle
15	Black	Tan	Blue	Blue	HMM Slow -> Fast TP
16	Black	Black	Blue	Black	Swimming Bout Duration Period 2 (sec), Swimming Bout Duration Period 4 (sec)
17	Black	Black	Tan	Black	Startle Magnitude Period 4, Swimming Bout Turning Angle Period 3

Table S22. Summary of mercury (Hg) significant treatment patterns found in Atlantic killifish behavior endpoints (Ctrl = control treatment, Tan = significant negative trend compared to control, Blue = significant positive trend compared to control, Black = no significant trend compared to control, HMM = Hidden Markov Chain model endpoint, TP = Transition Probability).

SCO-Ctrl vs SCO-Hg	Behavior Endpoint
Blue	Capture Attempt Ratio, Prey Capture Probability, Reaction Distance (mm), HMM Fast -> Fast TP, HMM Medium -> Medium TP
Tan	HMM Medium State Turning Angle Variation, HMM Medium -> Fast TP, Swimming Bout Duration Period 3 (sec), Total Time Swimming Period 3 (sec), Overall Step Length Period 4 (mm), Overall Step Length Variation Period 4, Total Distance Traveled Period 4 (mm)

Table S23. Summary of mercury (Hg) and PCB126 significant treatment patterns found in Scorton Creek (SCO) and PCB126 (PCB) effects on New Bedford Harbor (NBH) Atlantic killifish behavior endpoints (Ctrl = control treatment, Tan = significant negative trend compared to control, Blue = significant positive trend compared to control, Black = no significant trend compared to control, HMM = Hidden Markov Chain model endpoint).

	Significant Treatment Pattern					
Reference Number	SCO-Ctrl vs SCO-Hg	SCO-Ctrl vs SCO-PCB	SCO-Ctrl vs NBH-Ctrl	SCO-PCB vs NBH-PCB	NBH-Ctrl vs NBH-PCB	Behavior Endpoint
1	Tan	Tan	Black	Tan	Black	Total Time Swimming Period 3 (sec)
2	Blue	Blue	Blue	Black	Black	Capture Attempt Ratio
3	Tan	Tan	Black	Black	Black	Swimming Bout Duration Period 3 (sec)

4	Blue	Black	Blue	Black	Tan	HMM Medium -> Medium Transition Probabilities

Table S24. Significant PCB126 (PCB) treatment patterns shared by gene expression and behavior endpoints in the endpoints in Scorton Creek (SCO) and New Bedford Harbor (NBH) Atlantic killifish found in this study. Genes with unknown names and functions are reported in Table S19. Both the original and opposite behavior endpoint trends are listed (Ctrl = control treatment, Tan = significant negative trend compared to control, Blue = significant positive trend compared to control, Black = no significant trend compared to control, HMM = Hidden Markov Chain model endpoint).

Significant Treatment Pattern					Behavior Endpoint	
SCO-Ctrl vs SCO-PCB	SCO-Ctrl vs NBH-Ctrl	SCO-PCB vs NBH-PCB	NBH-Ctrl vs NBH-PCB	Gene Expression	Original Treatment Pattern	Opposite Treatment Pattern
Black	Black	Black	Tan	**Metabolic:** cmc2, rab4a	HMM Medium State Step Length Variation, Swimming Bout Turning Angle	HMM Fast -> Slow TP
Tan	Black	Black	Black	**Neural:** avp, si:dkey-175g6.2, uba1, gad2, ext2, usp22, spata2 **Nucleic:** polr2a, kdm2ab, rapgef1b, dyrk1b **Signaling:** pi4kab, plcl1, gareml, grin2ab, stx16, c2cd5, slc6a8, slc8a2b, kctd9a, prkab1a, si:ch211-168f7.5, slc30a1a **Metabolic:** arhgap1, mag, selenoi, epn3b, sucla2, plcxd3, elovl6, atp1b2b, arhgap25 **Development:** aldh1a2 **Circulatory:** b4gat1, pam, numb **Cellular:** ache, fam163ba, sec62, slc25a14, clptm1, coro7, bcat2, rusc1 **Protein Binding and Synthesis:** oat, znf598 **Miscellaneous:** abl2, klhl26, b3galt1b	Swimming Bout Duration Period 3 (sec), Total Distance Traveled (mm)	Overall Turning Angle Period 2

		**Neural:** grna, fam53b, psma6a, nusap1, scinla, pmm2, ckma **Nucleic:** nrm, anapc15, olig4, tead3b, msx1a, nsmce2, emx2, heyl, nt5c2l1, foxn4, rad51ap1, her12, pane1, cpsf3, pagr1, spi1b, ascl1b **Signaling:** myl1, adh5, si:dkey-148a17.6, fcer1g, mylz3, pvalb3, hvcn1, sparc **Metabolic:** naga, lcat, gch2, rgs18, rac2 **Development:** acta1b, tnnt3a, vegfd, dla **Sensory:** vps28, lhfpl4b, bco1 Stress: slc25a39, cpn1 **Circulatory:** hcls1, ckmb, mb **Transport:** scamp4, cahz **Cellular:** nmrk1, mlc1, egln3, mibp, hs2st1b, vsir, rdh8a, tmem45a, si:dkey-9i23.16 **Imunity:** ctss2.1, tnfaip8l2b **Protein Binding and Synthesis:** sumf1 **Miscellaneous:** si:dkey-225f5.4, si:ch211-236d3.4, fam89b	Overall Turning Angle Period 2	Swimming Bout Duration Period 3 (sec), Total Distance Traveled (mm)
		**Neural:** atcaya, ubap1, hectd1, rnf41, tulp4a, lrrc4.1, neurl1aa, desi1a, lnx1, sema3ab, zdhhc17, cntnap2a, usp24 **Nucleic:** fam98a, seta, senp3b, bhlhe41, rerea, rc3h1b, rprd2a, grid2ipa, evx2, khdc4, tent4a, kdm3b, arid2, fut9a, znf346, rfx1b, elk4, qkia, foxj3, srfb, zfr2, klf6a, larp4ab, pdik1l, ssbp4 **Signaling:** erbin, spred2a, crk, map3k9, ppp3ccb, nlk1, araf, gramd4a, ndrg3a, zmym2, bmp2k, slit1b, ppp2r5ca, iqsec2b, gpr63, pdpk1b, dusp8a, gnb1b **Metabolic:** tbc1d22b, gal3st3, arfgap1, casd1, atp8a2, cdk17, pitpnab, pdk3a, ralaa, ptdss1a, nudt3b **Development:** tmem65 Stress: rlim, kmt2e **Circulatory:** mybpc2b **Transport:** atp1a3a, ptpn23a, scamp1, slc6a17, ap2b1 **Cellular:** ano8b, zgc:114120, tmem86a, asphd2, si:dkeyp-27e10.3, shank1, enah, ubap2a, kiaa1549la, tm9sf3, syt14a, zdhhc20b, clip3, tspan7b, klc2, ubap2l, dmtn **Digestive:** mtor **Protein Binding and Synthesis:**	Total Time Swimming (sec)	Capture Attempt Ratio

		mcu, nlgn2a, bag6 **Miscellaneous:** ajm1, zgc:158464, scaf8		
		**Neural:** stmn1b, im:7136398, slc25a1b, exosc8, snapin **Nucleic:** acin1a, znf207b, pithd1, eif2a **Signaling:** rgn, micu2 **Metabolic:** gpx1a, hibadhb, chchd3b, rasd1, ntpcr, ptcd2 **Development:** acvrl1, psenen, fgfbp3 **Circulatory:** acta2 **Transport:** crabp1a, chmp5b, stxbp3 **Cellular:** ccdc90b, ppcs, c18h3orf33, cd63, srr, tha1, srxn1, tspan14, atp6ap1a, tbce, tmem9b, tspan3a **Digestive:** scpep1 **Imunity:** ifi30 **Protein Binding and Synthesis:** alg3	Capture Attempt Ratio	Total Time Swimming (sec)

Table S25. Individual based model results showing treatment means for individual larva survival and growth of Atlantic killifish *Fundulus heteroclitus* found in this study.

	IBM Output Mean	
Scenario	Survival (%)	Growth (mm/d)
Spring		
SCO-Ctrl	1.512	0.29871
SCO-MeHg	1.648	0.29782
SCO-PCB	0.044	0.12975
NBH-Ctrl	1.084	0.29197
NBH-PCB	0.416	0.29251
Summer		
SCO-Ctrl	1.068	0.30167
SCO-MeHg	1.244	0.30697
SCO-PCB	0.288	0.25546
NBH-Ctrl	0.788	0.28597
NBH-PCB	0.42	0.28322
